# STUB1-mediated K63-linked ubiquitination of UHRF1 promotes the progression of cholangiocarcinoma by maintaining DNA hypermethylation of PLA2G2A

**DOI:** 10.1186/s13046-024-03186-6

**Published:** 2024-09-13

**Authors:** Junsheng Chen, Da Wang, Guanhua Wu, Fei Xiong, Wenzheng Liu, Qi Wang, Yiyang Kuai, Wenhua Huang, Yongqiang Qi, Bing Wang, Yongjun Chen

**Affiliations:** 1 Department of Biliary-Pancreatic Surgery, Tongji Hospital, Tongji Medical College, Huazhong University of Science and Technology, 1095 Jiefang Avenue, Wuhan, Hubei 430074 China; 2 Department of General Surgery, Beijing Friendship Hospital, Capital Medical University, Beijing, 100050 China; 3 Department of Emergency, Tongji Hospital, Tongji Medical College, Huazhong University of Science and Technology, 1095 Jiefang Avenue, Wuhan, Hubei 430074 China; 4 Key Laboratory of Laparoscopic Technology of Zhejiang Province, Department of General Surgery, Sir Run-Run Shaw Hospital, Zhejiang University School of Medicine, Hangzhou, 310016 China

**Keywords:** STUB1, UHRF1, DNMT1, DNA methylation, PLA2G2A

## Abstract

**Background:**

Cholangiocarcinoma (CCA) is a highly malignant tumor characterized by a lack of effective targeted therapeutic strategies. The protein UHRF1 plays a pivotal role in the preservation of DNA methylation and works synergistically with DNMT1. Posttranscriptional modifications (PTMs), such as ubiquitination, play indispensable roles in facilitating this process. Nevertheless, the specific PTMs that regulate UHRF1 in CCA remain unidentified.

**Methods:**

We confirmed the interaction between STUB1 and UHRF1 through mass spectrometry analysis. Furthermore, we investigated the underlying mechanisms of the STUB1-UHRF1/DNMT1 axis via co-IP experiments, denaturing IP ubiquitination experiments, nuclear‒cytoplasmic separation and immunofluorescence experiments. The downstream PLA2G2A gene, regulated by the STUB1-UHRF1/DNMT1 axis, was identified via RNA-seq.  The negative regulatory mechanism of PLA2G2A was explored via bisulfite sequencing PCR (BSP) experiments to assess changes in promoter methylation. The roles of PLA2G2A and STUB1 in the proliferation, invasion, and migration of CCA cells were assessed using the CCK-8 assay, colony formation assay, Transwell assay, wound healing assay and xenograft mouse model. We evaluated the effects of STUB1/UHRF1 on cholangiocarcinoma by utilizing a primary CCA mouse model.

**Results:**

This study revealed that STUB1 interacts with UHRF1, resulting in an increase in the K63-linked ubiquitination of UHRF1. Consequently, this facilitates the nuclear translocation of UHRF1 and enhances its binding affinity with DNMT1. The STUB1-UHRF1/DNMT1 axis led to increased DNA methylation of the PLA2G2A promoter, subsequently repressing its expression. Increased STUB1 expression in CCA was inversely correlated with tumor progression and overall survival. Conversely, PLA2G2A functions as a tumor suppressor in CCA by inhibiting cell proliferation, invasion and migration.

**Conclusions:**

These findings suggest that the STUB1-mediated ubiquitination of UHRF1 plays a pivotal role in tumor progression by epigenetically silencing PLA2G2A, underscoring the potential of STUB1 as both a prognostic biomarker and therapeutic target for CCA.

**Graphical Abstract:**

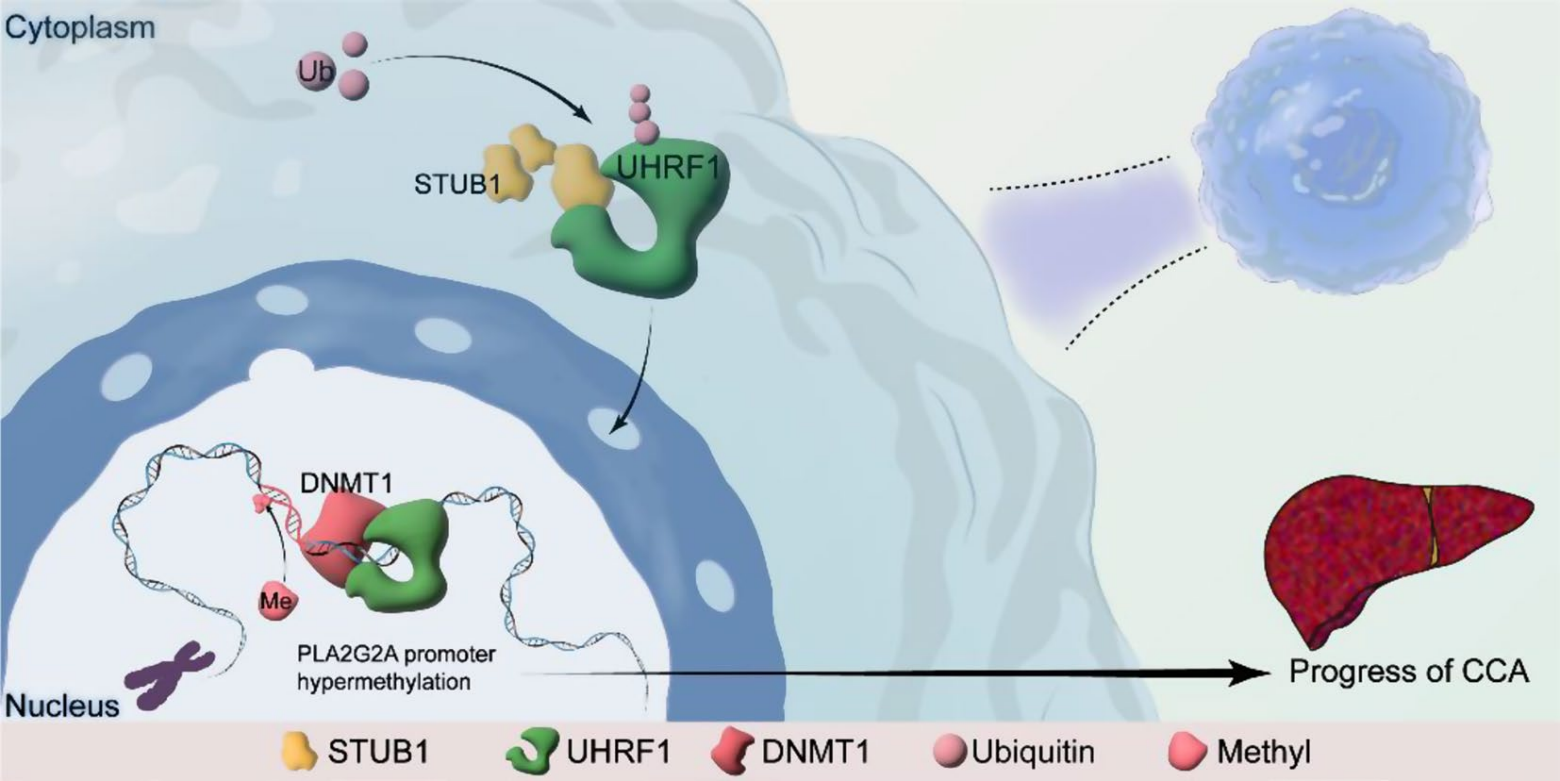

**Supplementary Information:**

The online version contains supplementary material available at 10.1186/s13046-024-03186-6.

## Background

Cholangiocarcinoma (CCA) is an exceptionally malignant tumor originating from the biliary epithelium. Surgical intervention serves as the primary curative option, although it is suitable for fewer than 30% of patients. Additionally, there is a high risk of cancer recurrence, and systemic therapy has limited effectiveness [1, 2]. Therefore, the quest for novel biomarkers pertaining to CCA-targeted molecular therapy is a pivotal task in tumor research.

The precise regulation of transcription plays a pivotal role in the development of all higher organisms. Posttranscriptional modifications (PTMs) intricately modulate the functionality of crucial proteins by impacting their stability, conformation, or subcellular localization, thereby influencing their physiological processes [3]. To date, more than 450 distinct protein modifications have been identified, including phosphorylation, acetylation, ubiquitination, SUMOylation and methylation [4]. The modification of proteins by ubiquitin typically involves enzymes that catalyze the activation of ubiquitin (E1s), its conjugation (E2s) and ligation to protein targets (E3s), as well as the participation of deubiquitinating enzymes [5, 6]. The K48-linked ubiquitination pathway is an extensively recognized mechanism for protein degradation, while the K63-linked ubiquitination pathway considerably contributes to the regulation of immune signaling, protein localization, and interactions associated with nondegradable functionalities [7–9]. The stability of the genome and normal gene expression are predominantly maintained by fixed or predetermined patterns of DNA methylation. Alterations in DNA methylation in cancer are considered promising for the development of robust diagnostic, prognostic, and predictive biomarkers [10, 11].

Human UHRF1 (also known as ICBP90) was initially discovered as a regulator of TopoIIalpha [12, 13], which is an oncogene that is overexpressed in a variety of solid and hematological tumors [14], including hepatocellular carcinoma [15], lung cancer [16, 17], and colorectal cancer [18]. The UHRF1-DNMT1 interaction is essential for the normal nuclear localization of DNMT1 and the maintenance of DNA methylation [19]. Studies have shown that the upregulation of UHRF1 expression or enhanced function promotes the epigenetic silencing of tumor suppressor genes (TSGs), thereby facilitating tumor progression and drug resistance in tumors [14, 20, 21]. Studies have shown that UHRF1 expression is increased in CCA and that higher levels of UHRF1 are associated with a poorer prognosis. UHRF1 also serves as an independent prognostic indicator in CCA patients. Moreover, reduced UHRF1 expression impedes the G1/S cell cycle transition, resulting in decreased cell proliferation under experimental conditions and inhibited tumor growth in vivo [22, 23]. The types of PTMs of UHRF1 include phosphorylation, ubiquitination, acetylation, glycosylation, and others, and PTMs of UHRF1 have been found to impact recruitment to DNMT1 and promote the maintenance of DNA methylation [24]. PTMs are essential in cancer research because they improve our understanding of cancer biology and help identify new diagnostic biomarkers and therapeutic targets. However, the regulatory mechanism of PTMs on UHRF1 in CCA and its effects on the biological function of CCA are still unclear.

STIP1 homology and U-Box containing protein 1 (STUB1), formerly known as CHIP (C-terminus of HSC70-Interacting Protein), is a chaperone-dependent E3 ubiquitin ligase that plays an essential role in various aspects of cancer, including tumorigenesis, progression, metastasis, drug resistance, and patient prognosis [25, 26]. The biological effects of STUB1 vary across different cancers, depending on the targeting of different substrates specific to each cancer type. STUB1 suppresses tumor progression by ubiquitinating and degrading various substrates, including HER2 [27], PGK1 [28], YAP [29], OCT4 [30], and EZH2 [30]. However, in kidney clear cell carcinoma, gallbladder cancer, and esophageal squamous cell carcinoma, it acts as a tumor-promoting factor. In certain cancers, such as colorectal cancer (CRC), glioma, and prostate cancer, STUB1 plays dual roles [25].

Phospholipase A2 group IIA (PLA2G2A) is a member of the phospholipase A2 (PLA2) family that is believed to play a role in regulating phospholipid metabolism in biomembranes. The involvement of PLA2G2A in tumor progression in humans has been demonstrated to be dualistic. High expression of PLA2G2A has been linked to shorter survival in patients with lung cancer [31] and prostate cancer [32]. Conversely, in gastric cancer, elevated expression of PLA2G2A is associated with improved patient survival. PLA2G2A expression is negatively correlated with infiltration depth, lymph node metastasis, and the tumor-lymph node-metastasis stage [33, 34]. Although there have been intriguing developments, the expression of PLA2G2A in CCA and its impact on prognosis have been discussed in only a limited number of studies [35]. Further validation is needed to understand the biological function of PLA2G2A in cholangiocarcinoma cells.

In this study, we discovered that STUB1 facilitates the K63-linked ubiquitination of UHRF1, thereby increasing its nuclear localization and binding affinity for DNMT1. Consequently, this leads to an increase in promoter DNA methylation of PLA2G2A mediated by UHRF1 and DNMT1, ultimately resulting in the inhibition of PLA2G2A expression. STUB1 actively participates in the regulation of PLA2G2A transcription through these PTMs and influences the progression of cholangiocarcinoma. The elucidation of these mechanisms and molecules in this study can offer valuable insights for the management of cholangiocarcinoma.

## Methods

### Patient samples

Human cholangiocarcinoma tissue samples were obtained from the Department of Biliary and Pancreatic Surgery at Tongji Hospital, Huazhong University of Science and Technology (Wuhan, China). These samples were surgically resected and confirmed as cholangiocarcinoma through pathological examination. Patients underwent postoperative follow-up until either their date of death or the last recorded follow-up. The collection procedures for all tissue samples were approved by the Ethics Committee of Tongji Hospital, Huazhong University of Science and Technology (HUST), in accordance with the principles outlined in the Declaration of Helsinki.

### Cell culture

Human cholangiocarcinoma cells (TFK1, HuCCT1) and human embryonic kidney cells (HEK293T) were cultured in RPMI 1640 or high-glucose DMEM medium (Servicebio, China) supplemented with 10% fetal bovine serum (NEWZERUM, New Zealand) at 37 °C and 5% carbon dioxide. All the cell lines were subjected to Mycoplasma infection testing and confirmed to be free of any infections.

### Plasmid construction

The full-length coding sequences (CDSs) of STUB1, UHRF1, and PLA2G2A were amplified from human cDNA via PCR and subsequently cloned and inserted into the pHAGE vector. Truncated fragments of UHRF1 and STUB1 were amplified via PCR via UHRF1-Flag and STUB1-Flag plasmids, respectively. Gene-specific small hairpin RNA primers were provided by Sangon Biotech Co., Ltd., and inserted into the pLKO.1 vector. The primer sequence information can be found in (Additional file 1).

### Viral packaging and viral infection

The target gene plasmid was cotransfected with the pMD2G and psPAX packaging plasmids into HEK293T cells. After 72 h of transient transfection using Polyethylene Linear (PEI) MW40000 (40816ES02, Yeasen, China), the supernatant was collected and filtered through a 0.45 μm filter (BS-PES-45, Biosharp, China). TFK1 and HuCCT1 cells were cultured in 1 mL of virus supernatant and 1 mL of RPMI 1640 complete medium supplemented with 2 µL of polybrene (40804ES76, Yeasen, China). After 24 h of transfection, puromycin hydrochloride (CL13900, Selleck, USA) was used for screening at a concentration of 1 µg/mL for 2 weeks. Then, Western blot experiments were conducted to assess the knockdown or overexpression efficiency of the target genes.

### Cell proliferation assay

A Cell Counting Kit-8 (CCK-8) (40203ES92, Yeasen, China) was used to assess cell viability. A total of 1000 stably transfected cells were seeded in 96-well plates and treated with the CCK-8 reagent working solution at different time points. The cells were incubated at 37 °C and 5% CO_2_ for 90 min, after which the absorbance was measured at a wavelength of 450 nm. For the colony formation experiment, 1000 cells were plated in 6-well plates with 2 mL of fresh culture medium and allowed to grow for 2 weeks. Staining was subsequently performed using a solution of 1% crystal violet to calculate the number of cell clones.

### Wound healing assay

The cells were evenly distributed in a 6-well plate, and scratches were induced using a 200 µL pipette tip after cell apposition. The scratched cells were subsequently removed by washing with PBS. Images were captured at 0 and 72 h after scratching, and the area of cell migration was quantified using ImageJ software.

### Transwell assay

To perform the invasion assay, we precoated Transwells with 100 mL of Matrigel at 37 ℃ for 1 h. Subsequently, 200 µL of serum-free medium containing 3 × 10^4^ cells was added to the center of each Transwell (polycarbonate membrane with a pore diameter of 8 μm; Corning Incorporated). The insert was then placed in a container filled with 800 µL of cell culture medium supplemented with 10% fetal bovine serum as the receiving well. Following incubation for either 48–72 h, the inserts were retrieved and stained with a solution of 1% crystal violet.

### Co-immunoprecipitation

The cells were transfected with the corresponding plasmids for 24 h, followed by cell lysis using IP buffer and the addition of complete protease inhibitors (REF: 04693132001, Roche, Indianapolis, IN). Ultrasound and centrifugation were performed on the cell lysate. A total of 40 µL of the supernatant from the cell lysis solution was used as the input, and the remaining portion was incubated with A/G magnetic beads (B26102, Biomake, USA) and the corresponding antibodies at 4 °C for 12 h. Subsequently, 5 rounds of washing of the magnetic beads with precooled IP buffer were performed for 5 min each. Finally, 40 µL of loading buffer was added to the magnetic beads, which were boiled at 95 °C for 15 min. The mixture was subsequently centrifuged, and the supernatant was collected for immunoblotting and subsequent analysis. IgG was used as a control for immunoprecipitation. The antibodies used are listed in (Additional file 2). The UHRF1 mass spectrometry binding protein obtained through co-immunoprecipitation is presented in (Additional file 3).

### GST pull-down assay

The CDS region of STUB1/UHRF1 was amplified via PCR and subsequently cloned and inserted into the pGEX-4T-1 vector. *Escherichia coli* BL21 were transformed with the resulting plasmid. Following the addition of IPTG (final concentration of 500 µM), bacterial cultivation was carried out at 16 °C for 12 h. Subsequently, the bacteria were lysed using GST buffer, followed by sonication and centrifugation to collect the supernatant. The supernatant was then incubated with GST magnetic beads on a rotating device at 4 °C for 1 h. Then, 5 washes with GST buffer for 5 min each were performed to remove nonspecific binding. The obtained GST magnetic beads were mixed with the cell lysate and incubated on a rotary shaker at 4 °C for 4 h. Following this step, the magnetic beads were washed 5 times with precooled IP buffer for 5 min each time. Finally, the magnetic beads were boiled in loading buffer at 95 ℃ for 15 min to elute bound proteins from the beads. The supernatant was collected after centrifugation and subjected to immunoblotting analysis.

### Nuclear and cytoplasmic protein purification and isolation

A Nuclear Protein and Cytoplasmic Protein Extraction Kit (P0028, Beyotime) was used to isolate the nuclear and cytoplasmic proteins. The cells were subsequently washed with PBS, and the cytoplasmic proteins were extracted using cytoplasmic protein extraction reagent A/B, whereas the nuclear proteins were extracted using nuclear protein extraction reagent. The proteins were subsequently subjected to analysis via SDS‒PAGE and immunoblotting.

### Western blot

Western blot analysis was performed by adding RIPA buffer supplemented with a cocktail of protease inhibitors to the lysed cell samples. Total protein extracts were mixed with loading buffer and denatured at 95 ℃ for 15 min. The proteins were subsequently separated via SDS‒PAGE and transferred onto a PVDF fiber membrane. The membrane was then blocked with 5% BSA at room temperature and incubated with primary antibodies at 4 ℃. Next, the membrane was incubated with the secondary antibody, and signal visualization was achieved via enhanced chemiluminescence.

### Total RNA isolation and RT‒qPCR

The cells were processed according to the experimental requirements. Total RNA was extracted using TRIzol (Vazyme, Nanjing, China), followed by reverse transcription of the RNA using HiScript^®^III RT SuperMix for qPCR (Vazyme). A quantitative analysis was subsequently performed using a PCR instrument that combines cDNA, SYBR Green dye (Vazyme), and the specific primers listed in (Additional file 4).

### Bisulfite sequencing PCR (BSP)

BSP primers were designed according to the online MethPrimer program (http://www.urogene.org/methprimer). The primers used can be found in (Additional file 5). DNA extraction was performed using a DNA extraction kit (D3396020000J12T006, Omega, USA), and bisulfite conversion for the BSP conversion reaction was carried out using a kit (EM101-02, Vazyme, China). PCR amplification was subsequently conducted using the Taq enzyme (C601-02, Vazyme, China). After purification of the PCR products, connection with the vector (EM101-02, Vazyme, China) was performed, and 10 bacterial clones were randomly selected for sequencing.

### Immunofluorescence staining

TFK1 and HuCCT1 cells were seeded on glass slides in 12-well plates. After 48 h, the cells were fixed with a 4% paraformaldehyde solution for 15 min and permeabilized with 0.1% Triton X-100 for 10 min. Subsequently, the cells were incubated overnight at 4 ℃ with primary antibodies, followed by incubation with secondary antibodies at room temperature for 1 h. Finally, cellular observations and imaging were performed using laser confocal microscopy. ImageJ software was used for further relative quantitative analysis, with the DAPI staining mask employed to define the regions of interest (ROIs) within the nucleus, thereby effectively distinguishing it from the cytoplasm. To eliminate artificial differences in staining intensity, we compared the staining intensities of the cytoplasm and nucleus to determine the cytoplasmic: nuclear ratio as a relative measure of UHRF1 nuclear localization [36]. The 3D Surface Plot plugin in ImageJ was used to visualize the relative fluorescence intensity of UHRF1 in the cytoplasm, where the luminance of the image was interpreted as the height of the plot [37]. The specific method can be accessed in the user guide for ImageJ (https://imagej.net/ij/plugins/surface-plot-3d.html).

### Experiments with animals

BALB/c (nu/nu) female nude mice and male C57BL/6J mice were procured from Jiangsu Jicui Yaokang Technology Co., Ltd. and housed in a specific pathogen-free facility at the Animal Center of Tongji Hospital, affiliated with Tongji Medical College, Huazhong University of Science and Technology.

For the xenograft experiments, 5-week-old female BALB/c (nu/nu) nude mice were randomly divided into groups (*n* = 8 in each group). A total of 3 × 10^6^ stably transduced cells were resuspended in 100 µL of PBS and injected into the dorsal side of each mouse. The experimental mice were euthanized by cervical dislocation, and the tumors were subsequently excised for further analysis.

To establish a mouse model of primary cholangiocarcinoma, we diluted 20 µg of pT3EF1aH-myr-Akt (179909, Addgene, USA) and 20 µg of pT3EF1aH-NICD1 (86500, Addgene, USA), along with 6 µg of the transposable plasmid pCMV (CAT) T7-SB100 (34879, Addgene, USA), in 2 mL of physiological saline. Subsequently, 2 mL from the plasmid mixture was injected into the lateral tail vein of male C57BL/6J mice (5 weeks old) within a time frame not exceeding 7 s. To overexpress the Uhrf1 and Stub1 proteins, we constructed the pT3EF1aH-Uhrf1/pT3EF1aH-Stub1 plasmid using the pT3EF1aH vector. An additional 20 µg of this construct plasmid was added to the aforementioned plasmid mixture. Liver samples were collected after hydrodynamic transfection for analysis of the occurrence and progression of CCA tumors.

### Immunohistochemical (IHC) staining

The detailed experimental procedures and protocols can be found in our previous research [38].

### Statistical analysis

The experiments were conducted independently and repeated at least three times. The data are presented as the means ± standard deviations unless otherwise specified. Statistical analysis was performed using GraphPad Prism Software 9.0, a widely recognized tool in scientific research. Student’s t test was used to compare two independent groups. Survival curve analysis was performed using the Kaplan‒Meier method and log rank test. The images were processed and integrated via ImageJ-win64, Photoshop and Adobe Illustrator 2022 for display. A *P* value < 0.05 was considered statistically significant: * *P* < 0.05, ** *P* < 0.01, *** *P* < 0.001.

## Results

### STUB1 directly interacts with UHRF1

**Fig. 1:**
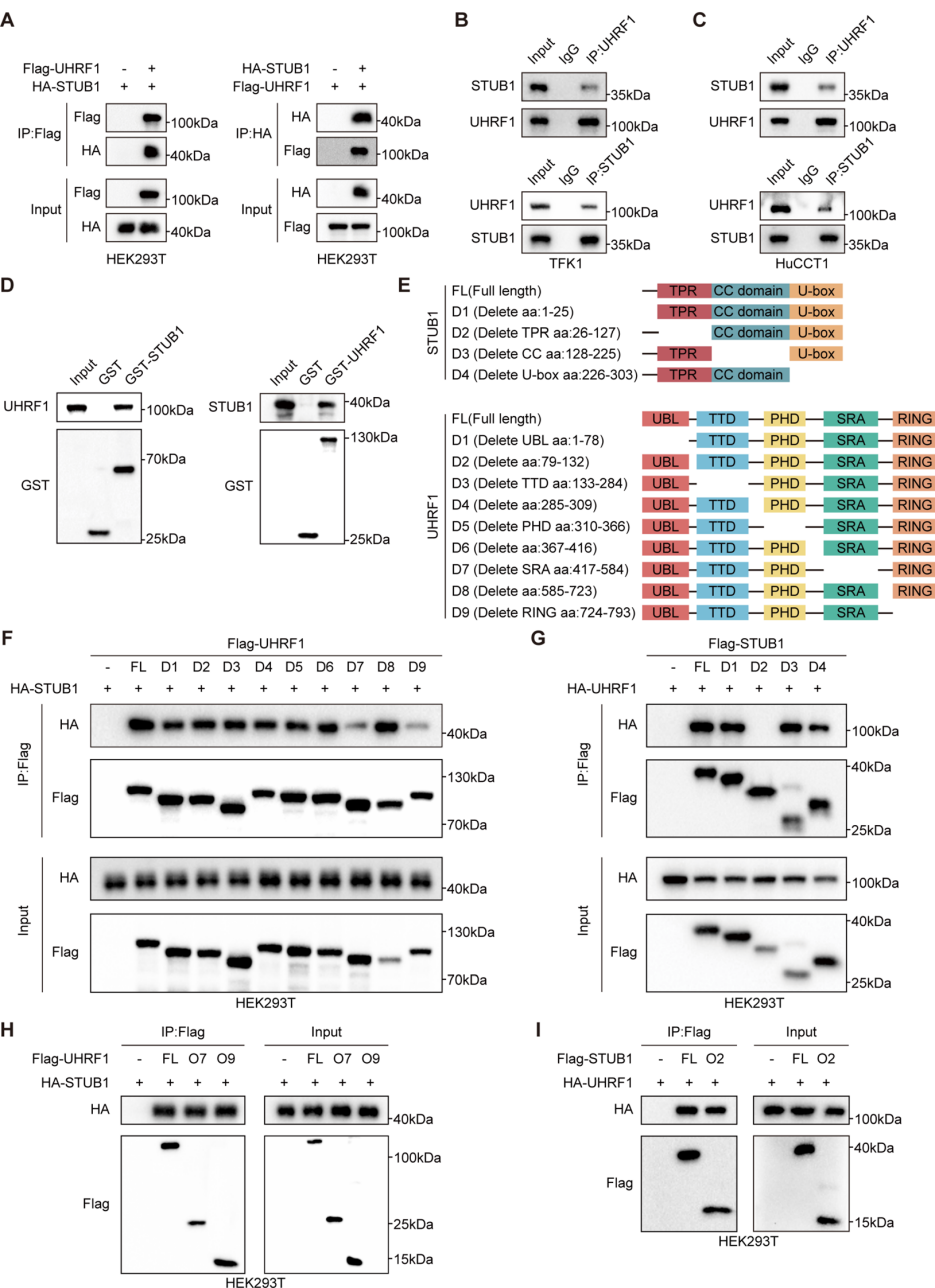
STUB1 directly interacts with UHRF1. **A**. Co-immunoprecipitation assays using Flag/HA antibody in HEK293T cells and immunoblotting to analyze the interaction of exogenous STUB1 with exogenous UHRF1. **B**, **C**. Co-immunoprecipitation assays using UHRF1/ STUB1 antibody in TFK1 (B) and HuCCT1 (C) cells and immunoblotting to analyze the interaction of endogenous STUB1 with endogenous UHRF1. **D**. The GST pull-down assay was conducted to identify the direct interaction between UHRF1 and STUB1. Cell lysates from HEK293T cells were incubated with GST, GST-STUB1, or GST‐UHRF1 conjugated to beads. The pull‐down samples and whole cell lysates were then analyzed using immunoblotting. **E**. Schematic diagram of the full-length and truncated fragments of UHRF1 and STUB1. **F**, **G**, **H**, **I**. HEK293T cells were transfected with full‐length or truncated fragments of UHRF1 or STUB1 as indicated. Cell lysates were collected and immunoprecipitated with anti‐Flag or anti‐HA antibodies to investigate the binding regions between UHRF1 and STUB1

To investigate the PTMs of UHRF1, we conducted co-immunoprecipitation (co-IP) experiments to isolate the UHRF1 protein and its interacting partners from HEK293T cells overexpressing UHRF1-Flag. The protein complexes were then analyzed via mass spectrometry, and the results revealed that the ubiquitin proteins that may interact with UHRF1 include STUB1, RNF2, TRIP12, and TRIM26 (Additional file 3). Subsequent co-IP experiments confirmed the binding of UHRF1 with only STUB1 and TRIM26 (Fig. 1A-C, Figure S1A-C). TRIM26 had no significant effect on the ubiquitination level of UHRF1 (Figure S1D). In addition, a direct interaction between UHRF1 and STUB1 was verified via a GST pull-down assay (Fig. 1D). To identify the domains where UHRF1 and STUB1 interact, we used several truncated forms of UHRF1 and STUB1 (Fig. 1E). The results revealed that the SRA domain (aa417-584) and the RING domain (aa724-793) of UHRF1 interact with STUB1 (Fig. 1F, H). Additionally, the TPR domain (aa26-127) of STUB1 was found to interact with UHRF1 (Fig. 1G, I).

### STUB1 promoted the K63-linked ubiquitination of UHRF1

**Fig. 2:**
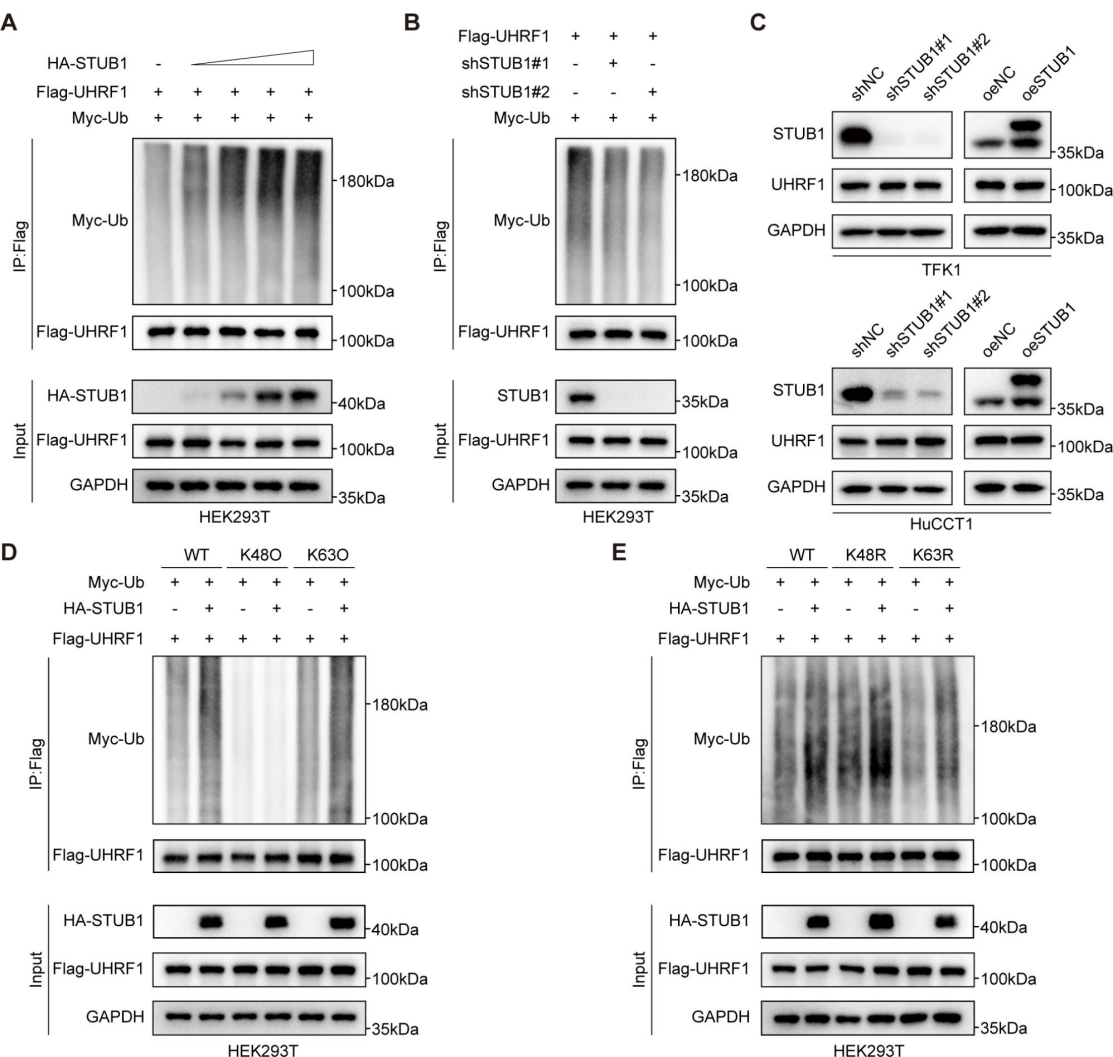
STUB1 promoted the K63-linked ubiquitination of UHRF1. **A** STUB1 (HA-tagged), ubiquitin (Myc-tagged), and UHRF1 (Flag-tagged) plasmids were exogenously overexpressed in HEK293T cells. Ubiquitination assays were conducted 48 h post-transfection, and the ubiquitination of UHRF1 was analyzed using a denaturing-immunoprecipitation assay. **B** Ubiquitination assays were conducted in HEK293T cells transfected with exogenously overexpressed ubiquitin (Myc-tagged) and UHRF1-(Flag-tagged) plasmids, along with shSTUB1. The ubiquitination of UHRF1 was then analyzed using a denaturing-immunoprecipitation assay. **C** Stable knockdown or overexpression of STUB1 by lentiviral transfection in TFK1 and HuCCT1 cells respectively, and immunoblotting detected the protein expression of STUB1 and UHRF1. **D**, **E**. HEK293T cells were transfected with plasmids overexpressing STUB1 (HA-tagged), Myc‐ubiquitin (wild type, K63R, K48R, K63O, K48O), and UHRF1 (Flag-tagged). Ubiquitination assays were conducted 48 h post-transfection, and the ubiquitination of UHRF1 was analyzed using a denaturing-immunoprecipitation assay

STUB1, which acts as an E3 ligase, was examined for its role in regulating the ubiquitination of UHRF1. Our study revealed an increase in UHRF1 protein ubiquitination, which was correlated with the expression of STUB1, as indicated by the results of the ubiquitination assay (Fig. 2A). In contrast, the knockdown of STUB1 inhibited the ubiquitination of the UHRF1 protein (Fig. 2B). To investigate whether STUB1 regulates UHRF1 protein stability, we knocked down and overexpressed STUB1 in CCA cells to assess the protein level of UHRF1. The results indicated that altering STUB1 expression did not affect the protein level of UHRF1 (Fig. 2C). To determine the specific type of STUB1-mediated UHRF1 polyubiquitin chain, we cotransfected HEK293T cells with UHRF1-Flag and Myc-ubiquitin (wild type, K48 only, or K63 only) in the presence or absence of STUB1-HA. The results of the ubiquitination assays demonstrated that STUB1 significantly increased the K63-linked polyubiquitination of UHRF1, with no notable effect on K48-linked polyubiquitination (Fig. 2D). Furthermore, ubiquitination assays conducted with the K63R mutant (ubiquitin with only the Lys63 to Arg mutation) revealed that the degree of ubiquitination of UHRF1 decreased compared with that of the wild-type ubiquitin molecule and the degree of ubiquitination of the K48R ubiquitin molecule (Fig. 2E). These findings suggest that STUB1 facilitates the K63-linked ubiquitination of UHRF1.

### K63-linked ubiquitination of UHRF1 promotes its nuclear localization and interaction with DNMT1

**Fig. 3:**
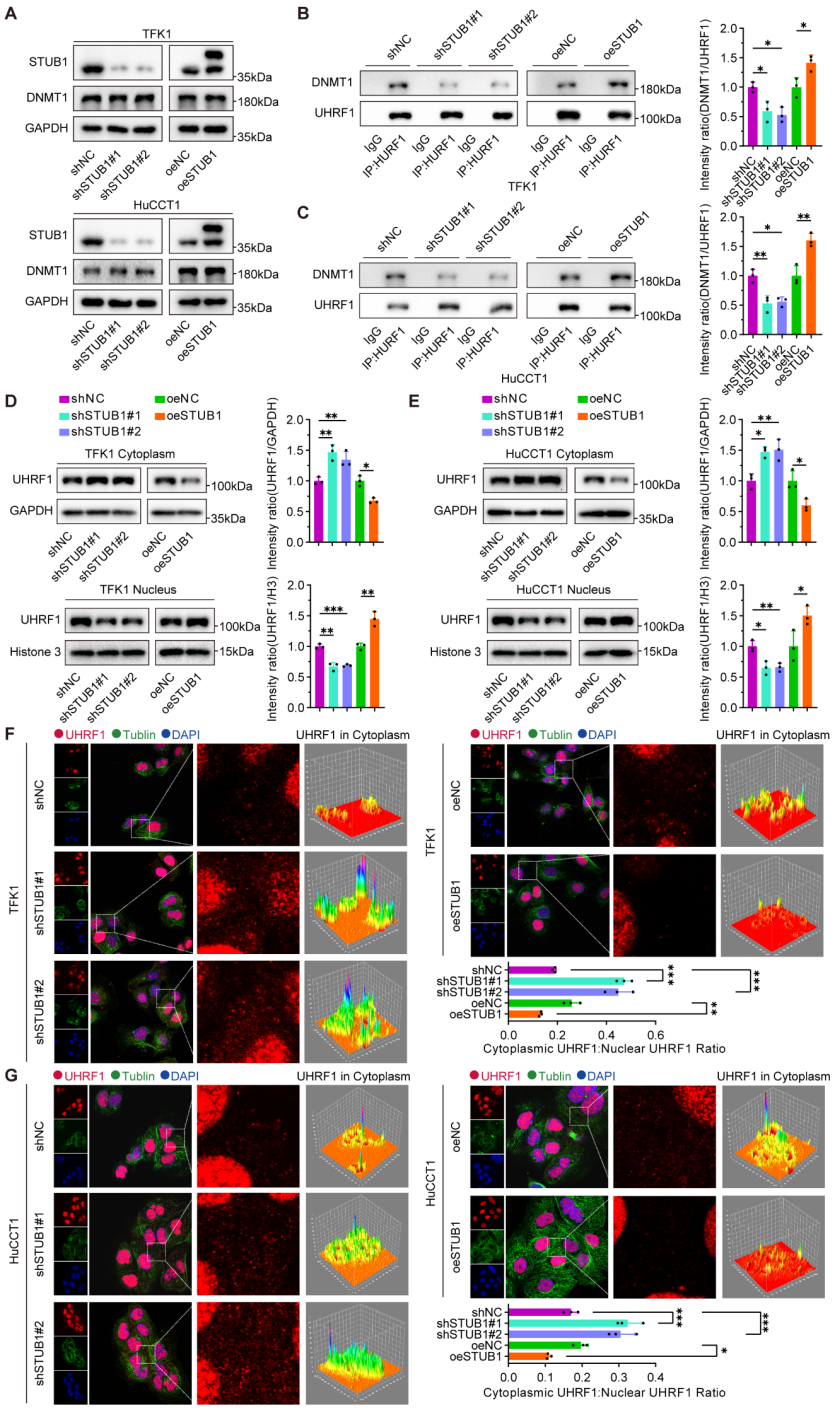
K63-linked ubiquitination of UHRF1 promotes its nuclear localization and interaction with DNMT1. **A**. Stable knockdown and overexpression of STUB1 by lentiviral transfection in TFK1 and HuCCT1 cells respectively and immunoblotting to detect the protein expression of STUB1 and DNMT1. **B**, **C**. Co-immunoprecipitation assays were conducted using UHRF1 antibody in TFK1 (B) and HuCCT1 (C) cells with stable knockdown and overexpression of STUB1 to analyze the UHRF1-DNMT1 interaction. **D**, **E**. Nuclear and cytoplasmic proteins were extracted from TFK1 (D) and HuCCT1 (E) cells with stable knockdown and overexpression of STUB1. Co-immunoprecipitation assays were then conducted to investigate the impact of UHRF1 distribution in the nucleus or cytoplasm. **F-G**. Immunofluorescence assays were performed to examine the distribution of UHRF1 in the cytoplasm of TFK1 (F) and HuCCT1 (G) cells with stable knockdown and overexpression of STUB1. The staining intensities of the cytoplasm and nucleus were compared to determine the cytoplasmic: nuclear ratio as a relative measure of UHRF1 nuclear localization. The 3D Surface Plot plugin in ImageJ was used to visualize the relative fluorescence intensity of UHRF1 in the cytoplasm, where the luminance of the image was interpreted as the height of the plot

Our results demonstrate that STUB1 enhances the K63-linked ubiquitination of UHRF1. Previous research has suggested that PTMs of UHRF1 play a crucial role in regulating its ability to recruit DNMT1 for DNA methylation processes [24]. Our experimental findings suggest that alterations in STUB1 expression do not lead to changes in the protein expression of DNMT1 in CCA cells (Fig. 3A). STUB1 was found to increase the recruitment of UHRF1 with DNMT1, as demonstrated by a co-IP assay (Fig. 3B-C). Through a nucleoplasmic separation assay, we observed that STUB1 facilitated the translocation of UHRF1 from the cytoplasm to the nucleus (Fig. 3D-E). Subsequent results from the cellular immunofluorescence assay indicated that depletion of STUB1 led to increased cytoplasmic distribution of UHRF1, whereas overexpression of STUB1 resulted in decreased cytoplasmic distribution of UHRF1 (Fig. 3F-G). These findings provide further evidence that STUB1 facilitates the K63-linked ubiquitination of UHRF1, promoting its nuclear localization and interaction with DNMT1.

### The STUB1-UHRF1/DNMT1 axis promotes epigenetic silencing of PLA2G2A in CCA cells

**Fig. 4:**
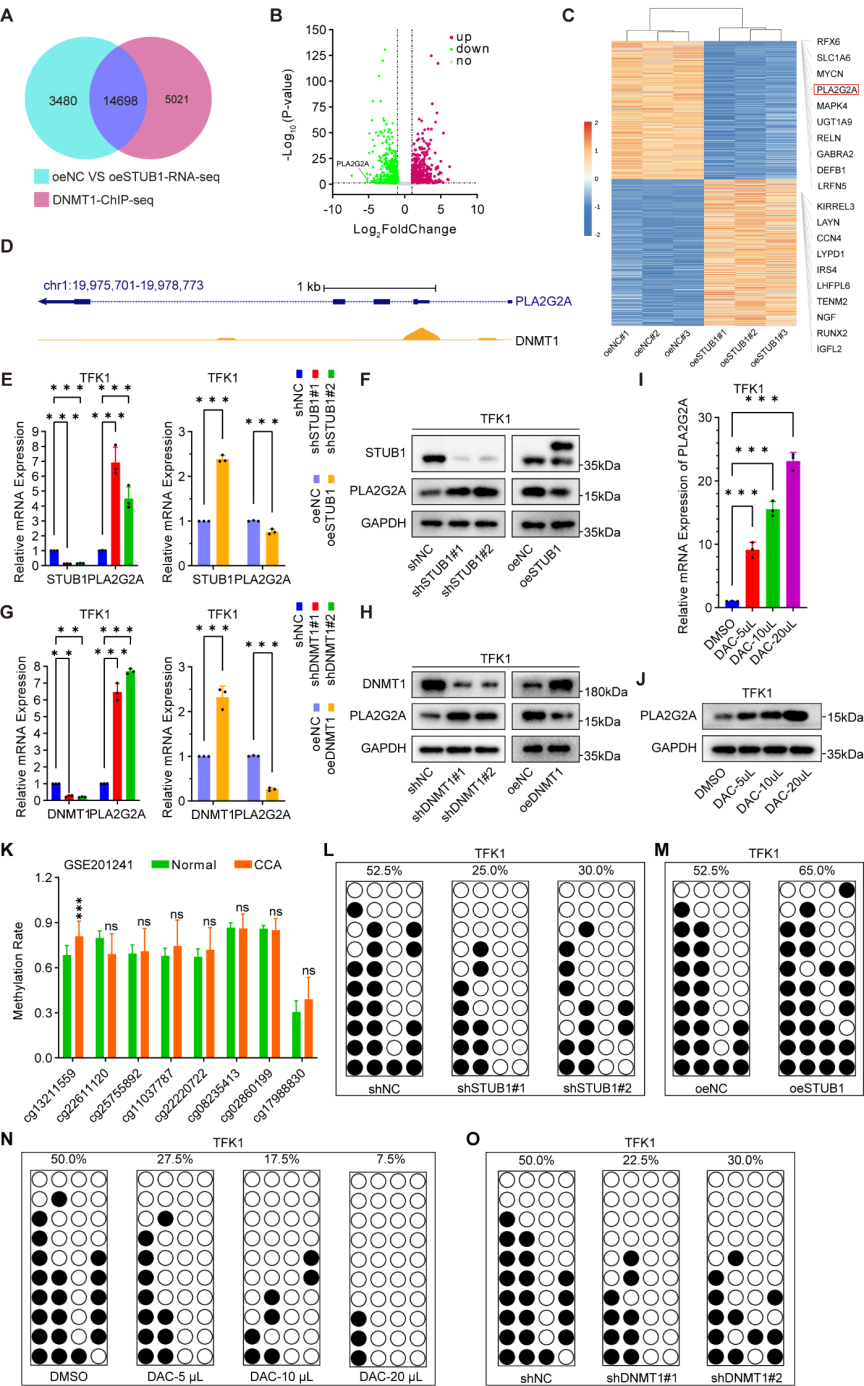
The STUB1-UHRF1/DNMT1 axis promotes epigenetic silencing of PLA2G2A in CCA cells. **A**. Venn diagram of the RNA-seq (negative control with overexpression of STUB1 in TFK1 cells) with ChIP-seq of DNMT1 from GEO database (GSE103331). **B**. The Volcano map of the RNA-seq (negative control with overexpression of STUB1 in TFK1 cells) with ChIP-seq of DNMT1 from GEO database (GSE103331). **C**. Heat map of the top 10 genes among down-regulated and up-regulated genes from the intersection of RNA-seq and ChIP-seq. **D.** ChIP-seq data from the GEO dataset (GSE103331) was used to analyze the distribution of DNMT1 in the PLA2G2A promoter. **E**, **G**. RNA was extracted from TFK1 cells with stable knockdown and overexpression of STUB1 (E) and DNMT1 (G) for RT-qPCR analysis to assess mRNA levels of PLA2G2A. **F**, **H**. Proteins were extracted from TFK1 cells with stable knockdown and overexpression of STUB1 (F) and DNMT1 (H) for Western blotting experiments to assess the protein expression of PLA2G2A. **I**, **J**. The mRNA and protein expression of PLA2G2A in TFK1 cells treated with DAC were analyzed using RT-qPCR (I) and Western blotting (J) assays. **K**. The DNA methylation of PLA2G2A promoter in CCA and para-carcinoma normal tissues was analyzed by a GEO dataset (GSE201241). **L-O**. DNA methylation of the PLA2A2G promoter was assessed using the BSP assays in TFK1 cells with stable knockdown of STUB1 (L), stable overexpression of STUB1 (M), DAC treatment (N), and stable knockdown of DNMT1 (O)

UHRF1/DNMT1-mediated DNA methylation plays a crucial role in promoting the epigenetic silencing of TSGs and facilitating tumor progression [14]. To investigate the specific TSGs regulated by the STUB1-UHRF1/DNMT1 axis in CCA cells, RNA-seq analysis of overexpressed STUB1 and negative control TFK1 cells was performed (GSE275480). In addition, we compared the RNA-seq results with the ChIP-seq results for DNMT1 from the GEO database (Fig. 4A-B). We then sorted the top ten genes (*p* < 0.05) that were downregulated or upregulated on the basis of log2FC values, and the results are shown in Fig. 4C. Since DNA methylation usually leads to transcriptional silencing, we focused on the top 10 genes in the downregulated group. RT‒qPCR was performed, and the results revealed that only PLA2G2A was significantly inhibited after the overexpression of STUB1 (log2FC=-5.18, *p* < 0.05, ranked 4th among the downregulated genes) (Figure S2A). In addition, we determined the distribution of DNMT1 in the PLA2G2A promoter via ChIP-seq of the online database (Fig. 4D). STUB1 knockdown was observed to increase the transcription of PLA2G2A, whereas STUB1 overexpression was found to suppress the transcription of PLA2G2A (Fig. 4E-F, Figure S2B-C). Similarly, DNMT1 knockdown was shown to stimulate the transcription of PLA2G2A, whereas DNMT1 overexpression was demonstrated to inhibit the transcription of PLA2G2A (Fig. 4G-H, Figure S2D-E). In addition, the transcription of PLA2G2A was increased by the DNA methyltransferase inhibitor decitabine (DAC) (Fig. 4I-J, Figure S2F-G). Additionally, our analysis revealed that DNA methylation at the PLA2G2A promoter at the cg13211559 site was greater in CCA tissues than in paracarcinoma normal tissues, as shown by online data analysis (Fig. 4K). Therefore, the primers for the BSP assays were specifically designed for this locus to detect the corresponding DNA methylation. The findings revealed that the knockdown of STUB1 inhibited the promoter DNA methylation of PLA2G2A (Fig. 4L), whereas overexpression of STUB1 promoted the promoter DNA methylation of PLA2G2A (Fig. 4M). DAC treatment inhibited promoter DNA methylation of PLA2G2A, and the level of promoter DNA methylation of PLA2G2A was correlated with the concentration of DAC in CCA cells (Fig. 4N). Furthermore, in CCA cells, the knockdown of DNMT1 inhibited the promoter DNA methylation of PLA2G2A (Fig. 4O). These findings indicate that the STUB1-UHRF1/DNMT1 axis plays a role in promoting the silencing of PLA2G2A through DNA methylation in CCA cells.

### PLA2G2A inhibited the proliferation, invasion, and migration of CCA cells

**Fig. 5:**
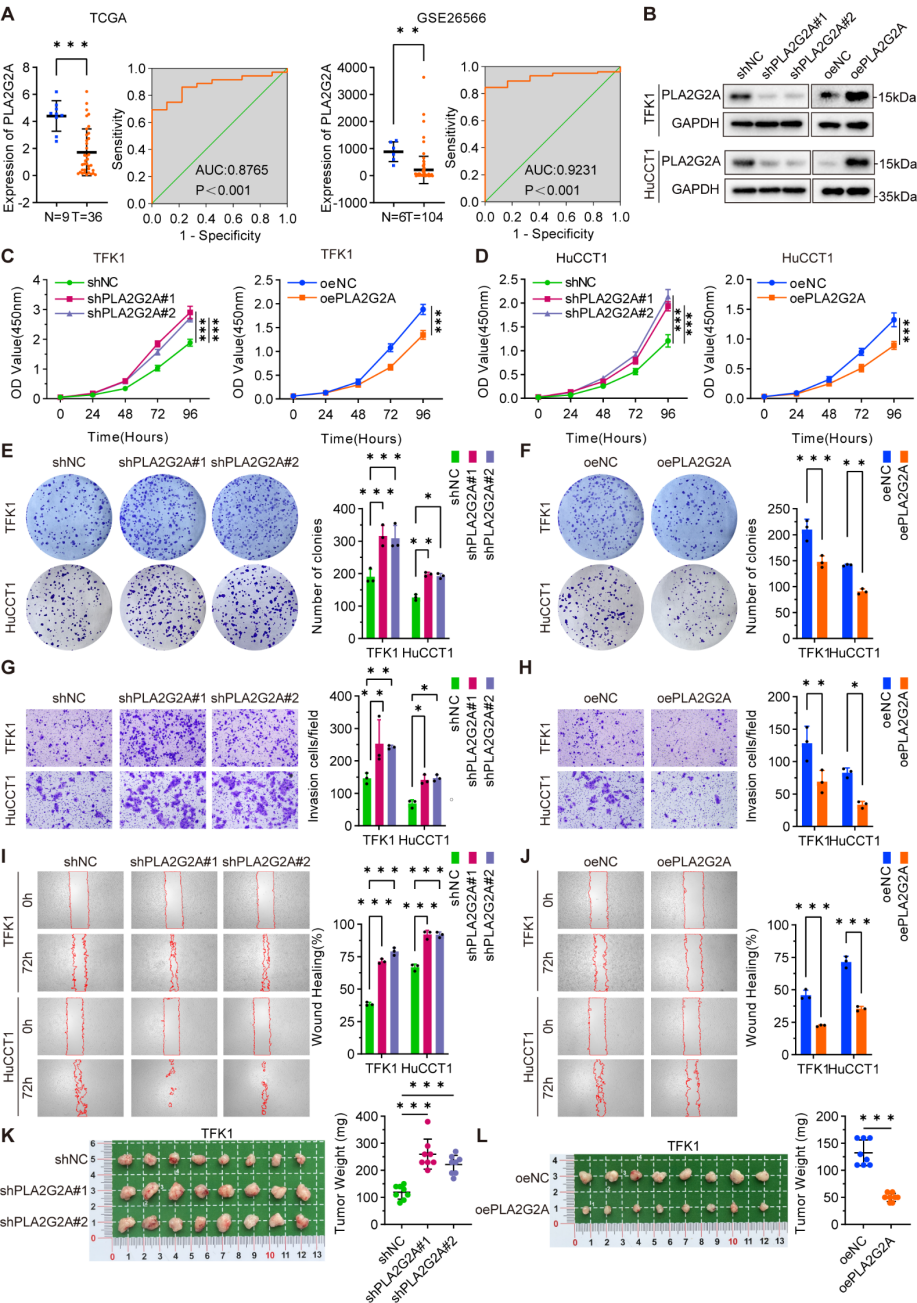
PLA2G2A inhibited the proliferation, invasion, and migration of CCA cells. **A.** The mRNA expression of PLA2G2A in CCA and para-carcinoma normal tissues was analyzed using TCGA and GEO dataset (GSE26566), and the diagnostic utility of the data was demonstrated through receiver operating characteristic (ROC) curves. **B.** Stable knockdown and overexpression of PLA2G2A were achieved through lentiviral transfection in TFK1 and HuCCT1 cell lines respectively and protein levels of PLA2G2A were then detected using Western blotting assays. **C**, **D**. The proliferative capacity of stable knockdown and overexpression of PLA2G2A in TFK1 (C) and HuCCT1 (D) cells was assessed using CCK-8 assays. **E**, **F**. Stable knockdown (E) and overexpression (F) of PLA2G2A in TFK1 and HuCCT1 cells were evaluated in plate cloning assays, with representative plots on the left and statistical analysis on the right. **G**, **H**. The invasive capacity of stable knockdown (G) and overexpression (H) of PLA2G2A in TFK1 and HuCCT1 cells was evaluated in Transwell assays, with representative plots on the left and statistical analysis on the right. **I**, **J**. the migratory capacity of stable knockdown (I) and overexpression (J) of PLA2G2A in TFK1 and HuCCT1 cells was evaluated in wound healing assays, with representative plots on the left and statistical analysis on the right. **K**, **L**. Xenograft tumor model of TFK1 cells stably knocking down (K) and overexpressing (L) PLA2G2A, general view of tumors on the left, statistical analysis of tumor weights on the right

PLA2G2A has been identified as a tumor suppressor in gastric cancer, where it inhibits the invasion and migration of cancer cells [33]. However, further research is needed to confirm its role in CCA cells. Initially, we observed a decrease in the mRNA expression of PLA2G2A in CCA tissues compared with that in paracarcinoma normal tissues through online database analysis (Fig. 5A). We subsequently performed knockdown and overexpression of PLA2G2A cell lines in CCA cells for further phenotypic validation (Fig. 5B). The results demonstrated that reducing PLA2G2A levels increased the proliferative capacity of CCA cells, as indicated by the results of the CCK-8 and plate cloning assays (Fig. 5C-D, E), whereas the overexpression of PLA2G2A suppressed the proliferative capacity of the cells (Fig. 5C-D, F). Additionally, Transwell and wound healing assays revealed that the knockdown of PLA2G2A enhanced the invasive and migratory capabilities of CCA cells (Fig. 5G, I), whereas the overexpression of PLA2G2A inhibited these abilities (Fig. 5H, J). In further in vivo assays, downregulation of PLA2G2A promoted the growth of CCA cell xenograft tumors (Fig. 5K), whereas overexpression of PLA2G2A inhibited CCA cell xenograft tumor growth (Fig. 5L). Therefore, as a tumor suppressor in CCA cells, PLA2G2A restrains proliferation, invasion, and migration.

### Upregulation of STUB1 is correlated with aggressive phenotypes and poor prognosis in CCA

**Fig. 6:**
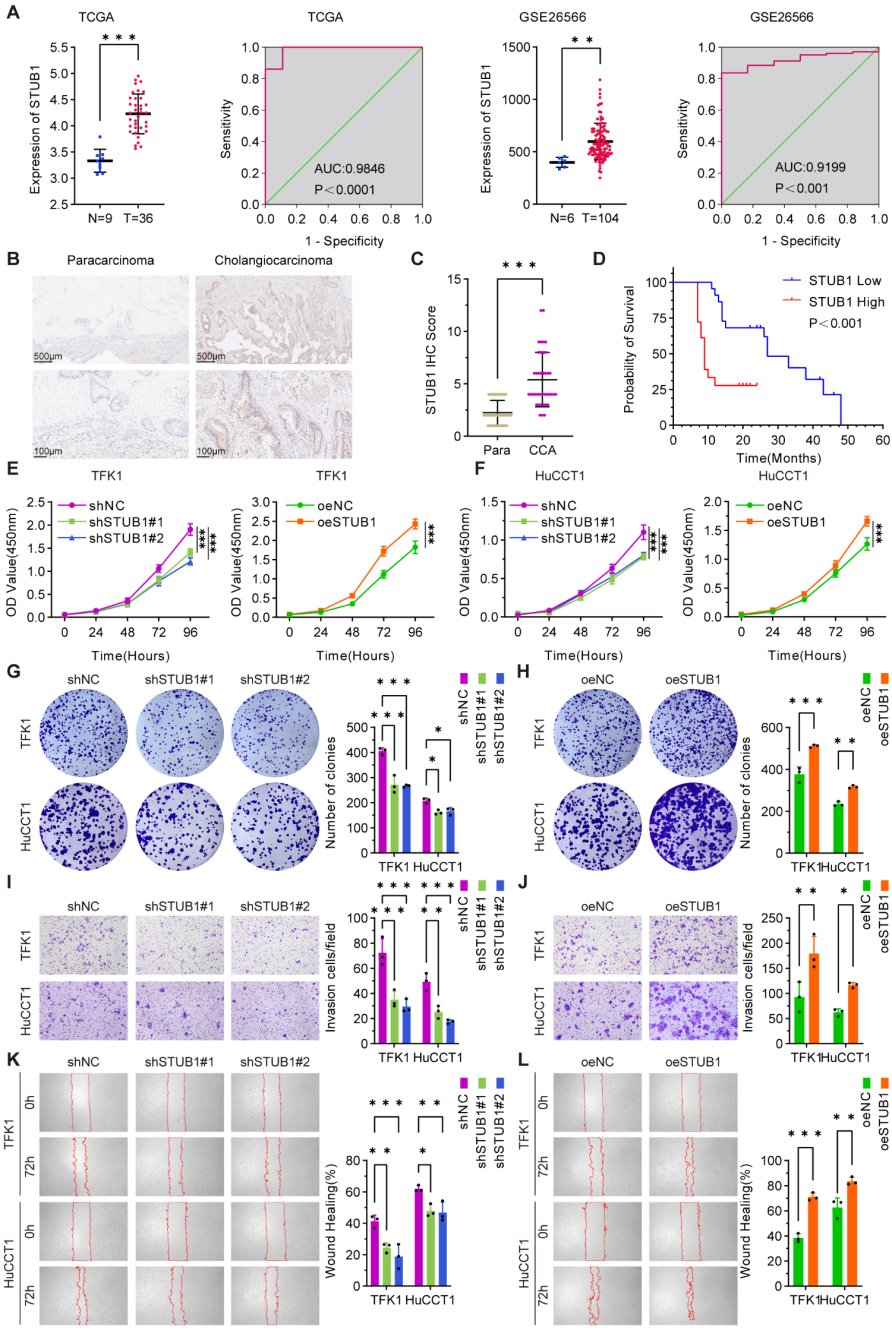
Upregulation of STUB1 is correlated with aggressive phenotypes and poor prognosis in CCA. **A**. The analysis of mRNA levels of STUB1 in CCA and para-carcinoma normal tissues was conducted using TCGA and GEO dataset (GSE26566) (left), along with the receiver operating curve (ROC) (right). **B**, **C**. IHC experiments were performed to analyze STUB1 expression in CCA and para-carcinoma normal tissues, with a representative graph (B) and statistical analysis of IHC scores (C). The study included 40 para-carcinoma and 40 CCA samples. **D**. Survival analysis curves (overall survival) were generated for CCA patients with high versus low expression of STUB1 in CCA tissues. The group consisted of 22 patients with low expression of STUB1 and 18 patients with high expression of STUB1. **E**, **F**. The proliferative capacity of stable knockdown and overexpression of STUB1 in TFK1 (E) and HuCCT1 (F) cells was assessed using CCK-8 assays. **G**, **H**. Stable knockdown (G) and overexpression (H) of STUB1 in TFK1 and HuCCT1 cells were evaluated in plate cloning assays, with representative plots on the left and statistical analysis on the right. **I**, **J**. The invasive capacity of stable knockdown (I) and overexpression (J) of STUB1 in TFK1 and HuCCT1 cells was evaluated in Transwell assays, with representative plots on the left and statistical analysis on the right. **K**, **L**. The migratory capacity of stable knockdown (K) and overexpression (L) of STUB1 in TFK1 and HuCCT1 cells was evaluated in wound healing assays, with representative plots on the left and statistical analysis on the right

The biological role of STUB1 in CCA was further investigated. An online database analysis revealed that the expression of STUB1 was greater in CCA tissues than in paracarcinoma normal tissues (Fig. 6A). Subsequent validation through IHC experiments revealed that the expression of STUB1 was greater in CCA tissues than in paracarcinoma normal tissues (Fig. 6B-C; Table 1). Furthermore, high expression of STUB1 in CCA patients was correlated with a poor prognosis (Fig. 6D). CCK-8 and plate colony formation assays revealed that the knockdown of STUB1 hindered the proliferative capacity of CCA cells (Fig. 6E-F, G), whereas the overexpression of STUB1 increased the proliferative ability of CCA cells (Fig. 6E-F, H). Additionally, Transwell and wound healing assays revealed that the knockdown of STUB1 suppressed the invasive and migratory capabilities of CCA cells (Fig. 6I, K), whereas the overexpression of STUB1 increased the invasive and migratory capacities of CCA cells (Fig. 6J, L). Overall, as a tumor promoter, STUB1 facilitates the proliferation, invasion, and migration of CCA cells.

**Table 1:** Clinicopathologic characteristics

Clinical characteristics	Numbers of patients	STUB1		*P*-Value
		Low expression	High expression	
Age (years)				0.19
≤ 60	14	10	4	
>60	26	12	14	
Gender				0.50
Male	29	17	12	
Female	11	5	6	
BMI				0.99
< 24	30	16	14	
≥ 24	10	6	4	
Differentiation				**0.02**
I^*^	24	17	7	
II^*^	16	5	11	
Stage				0.32
I and II	28	17	11	
III and IV	12	5	7	

Low expression: IHC staining index < 6; High expression: IHC staining index ≥ 6

I^*^: Well differentiation + Well to moderately differentiation + Moderately differentiation

II^*^: Moderately to low differentiation + Low differentiation

### STUB1 promotes the progression of CCA in vivo

**Fig. 7:**
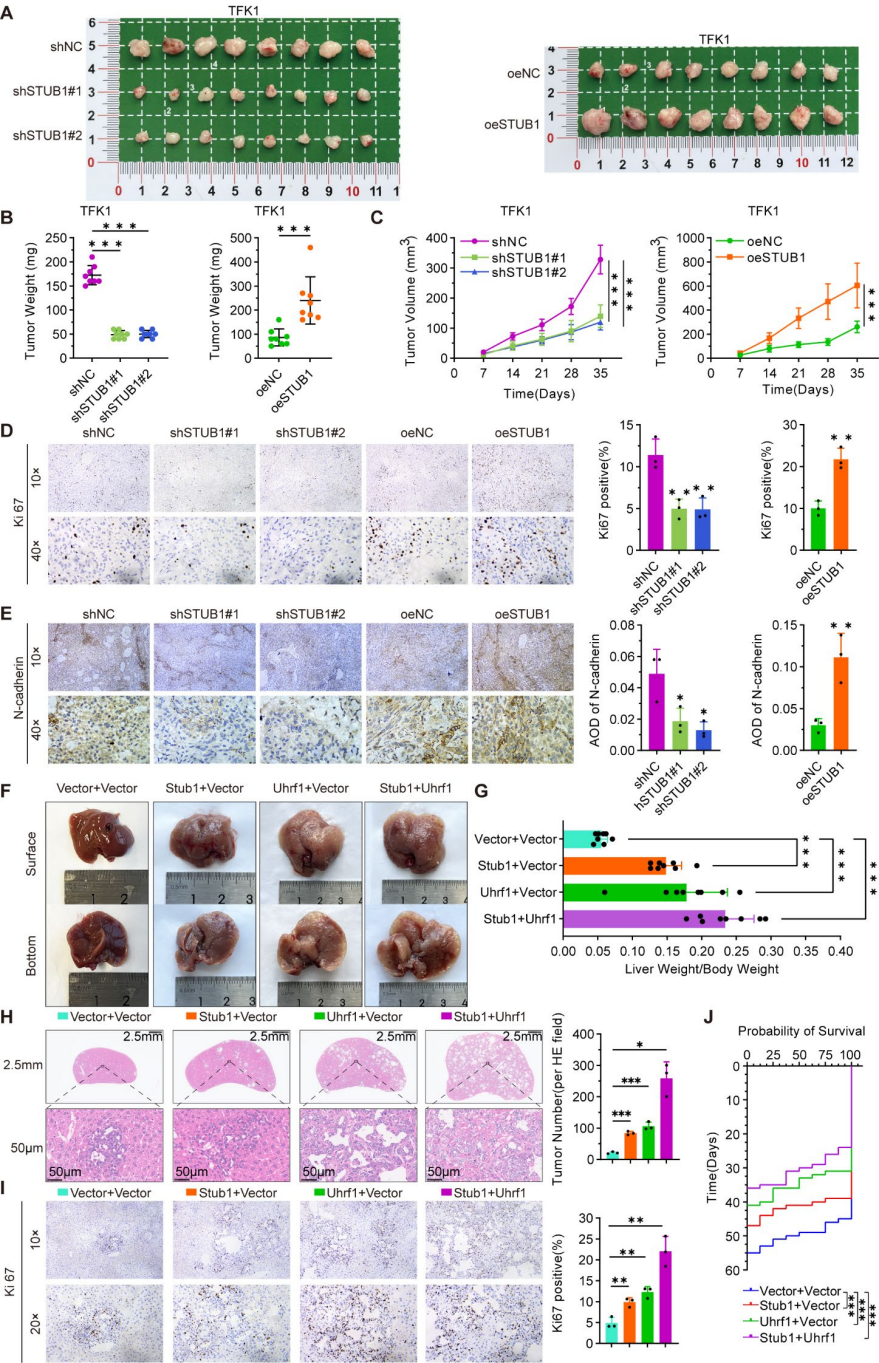
STUB1 promotes the progression of CCA in vivo. **A-C**. Xenograft tumor model of TFK1 cells was established with stable knockdown and overexpression of STUB1. The results included a general view of the tumor (A), statistical analysis of tumor weight (B), and assessment of tumor growth volume (C). **D**, **E**. IHC assays were conducted to analyze the expression of Ki67 (D) and N-cadherin (E) in tumor tissues, with representative plots on the left and statistical analysis on the right. **F**. Overview of tumor burden in mouse livers with Vector + Vector, Vector + Stub1, Vector + Uhrf1 and Stub1 + Uhrf1. **G**. Ratios of liver weight to body weight in Vector + Vector, Vector + Stub1, Vector + Uhrf1 and Stub1 + Uhrf1 mouse. **H**. HE-staining images of livers in Vector + Vector, Vector + Stub1, Vector + Uhrf1 and Stub1 + Uhrf1 mice (Left: Representative HE-staining images. Right: Quantitative analysis of tumor number). **I**. IHC assays detected of Ki67 in the tumors (Left: Representative images. Right: Quantitative analysis). **J**. Survival curve of Vector + Vector, Vector + Stub1, Vector + Uhrf1 and Stub1 + Uhrf1 mice

In vivo assays revealed that reducing the expression of STUB1 hindered the growth of CCA cell xenograft tumors, whereas increasing the expression of STUB1 increased tumor growth (Fig. 7A). This was evidenced by a decrease in tumor weight and growth volume upon knockdown of STUB1 and an increase in weight and volume with overexpression of STUB1 (Fig. 7B-C). Knockdown of STUB1 was observed to decrease the expression of Ki67 and N-cadherin in tumor cells, as determined by an IHC assay. Conversely, the overexpression of STUB1 increased the expression of Ki67 and N-cadherin in tumor cells (Fig. 7D-E). In the mouse primary CCA model, the overexpression of Stub1 and Uhrf1 individually promoted tumor progression, whereas the combined overexpression of both Stub1 and Uhrf1 further enhanced tumor progression (Fig. 7F), as demonstrated by the higher liver weight-to-body weight ratios observed in each group (Fig. 7G). HE staining of the liver revealed that Stub1 and Uhrf1 increased the number of lesions in the tumor (Fig. 7H). Additionally, IHC assays demonstrated that Stub1 and Uhrf1 enhanced the expression of Ki67 in tumor cells (Fig. 7I). Survival analysis indicated that the overexpression of Stub1 and Uhrf1 led to a poorer prognosis, with the worst prognosis observed in cases with simultaneous overexpression of Stub1 and Uhrf1 (Fig. 7J). Furthermore, immunohistochemical analysis of these animal models revealed that STUB1 facilitates the nuclear localization of UHRF1, while both STUB1 and UHRF1 exert inhibitory effects on the expression of PLA2G2A (Figure S3A-B).

## Discussion

DNA methylation is an important epigenetic modification that directly affects gene transcription [39]. DNA methylation plays an important role in the normal growth and development of organisms [40] and is involved in several pathological conditions, such as the genesis and development of cancer [41]. DNMT1 is a core enzyme that maintains intracellular DNA methylation, whereas UHRF1, a cofactor of DNMT1, recruits DNMT1 to the hemi-methylation site and plays an important role in the regulation of DNA methylation [42, 43]. DNMT1 was initially found to maintain DNA methylation in the genome, and in recent years, DNMT1 has also been shown to function in de novo DNA methylation at specific developmental stages [44, 45]. Aberrant overexpression of UHRF1 is observed in a number of types of cancer, and the overexpression of UHRF1 suppresses the transcription of TSGs through DNMT1-mediated DNA methylation [46]. In addition, PTMs of UHRF1 are involved in aberrant DNA methylation in cancer. For example, HDAC1 deacetylates UHRF1 at the K490 site, which allows UHRF1 to bind hemimethylated DNA in the S phase and be recruited to chromatin, thus allowing the DNA methylation process to proceed correctly [47].

Ubiquitination plays a crucial role in regulating protein degradation, subcellular localization, and protein‒protein interactions, impacting a wide range of cellular processes, such as signal transduction, cell cycle progression, DNA repair, and the immune response [48]. UHRF1 can be ubiquitinated by the E3 ubiquitin ligase SCF (β-TrCP), which in turn promotes its proteasomal degradation [49]. USP7 plays a crucial role in safeguarding UHRF1 from proteasomal degradation by binding to its SRA domain via the TRAF-like domain. This interaction is vital for preserving DNA methylation. Recent studies have indicated that USP7 not only facilitates the deubiquitination of UHRF1 but also forms a complex with it, influencing the role of UHRF1 in chromatin remodeling [50].

Our findings indicated that STUB1 interacts with the SRA and RING domains of UHRF1 via the TPR domain. As an E3 ubiquitin ligase, STUB1 promotes the K63-linked ubiquitination of UHRF1. Interestingly, the STUB1-mediated ubiquitination of UHRF1 does not impact the protein stability of UHRF1. Instead, it enhances the translocation of UHRF1 from the cytoplasm to the nucleus and facilitates its interaction with DNMT1. Increased UHRF1 entry into the nucleus and binding to DNMT1 also promoted the DNA methylation function of DNMT1. Transcriptome sequencing analysis and validation revealed that the overexpression of STUB1 repressed the expression of PLA2G2A. Subsequent bioinformatics analysis revealed that DNA methylation of PLA2G2A was greater in CCA tissue than in paracarcinoma normal tissue. This study confirmed that STUB1 regulates PLA2G2A expression via DNA methylation. Specifically, STUB1 was found to increase PLA2G2A promoter DNA methylation. Knocking down DNMT1 and treatment with a DNA methyltransferase inhibitor (DAC) resulted in increased PLA2G2A expression and inhibited promoter DNA methylation in CCA cells. STUB1 facilitates DNA methylation and suppresses PLA2G2A transcription via the involvement of UHRF1 and DNMT1. These findings suggest that the STUB1-UHRF1/DNMT1 axis plays a crucial role in the epigenetic silencing of PLA2G2A in CCA cells.

Previous studies have demonstrated that PLA2G2A functions as a tumor suppressor gene in esophageal squamous cell carcinoma [51] and gastric cancer [33]. Furthermore, PLA2G2A has been shown to impede colorectal tumor growth in mice [52]. Our in vitro and in vivo assays also revealed that the overexpression of PLA2G2A suppresses the proliferation, invasion, and migration of CCA cells. These findings suggest that PLA2G2A acts as a tumor suppressor gene in CCA cells, which is supported by the observed reduction in the expression of PLA2A2G in CCA tissues. The biological effects of STUB1 vary across different types of cancers. The functions of STUB1 as an oncogene in non-small cell lung, breast, renal cell, oral, gastric, head and neck, and pancreatic cancers. Conversely, the overexpression of STUB1 has been linked to negative outcomes and tumor advancement in certain cases, such as renal clear cell, gallbladder, and esophageal squamous cell carcinoma [25]. Our findings indicate that STUB1 expression is elevated in CCA and that patients with high STUB1 expression have a poorer prognosis. Functional assays revealed that STUB1 plays a role in enhancing the proliferation, invasion, and migration of CCA cells, thereby influencing the development of primary CCA in mice. These results suggest that monitoring STUB1 expression could serve as a valuable prognostic tool and potential target for therapeutic interventions in CCA.

Overall, our findings demonstrate that the STUB1-UHRF1/DNMT1 axis plays a critical role in driving the progression of CCA by silencing PLA2G2A through epigenetic mechanisms. STUB1 facilitates the K63-linked ubiquitination of the UHRF1 protein, increasing its nuclear translocation and recruitment of DNMT1, ultimately leading to DNA methylation of the PLA2G2A promoter. The expression of STUB1 may have clinical implications as a prognostic marker and potential therapeutic target in CCA.

## Conclusion

These findings suggest that the STUB1-mediated ubiquitination of UHRF1 plays a pivotal role in tumor progression by epigenetically silencing PLA2G2A, underscoring the potential of STUB1 as both a prognostic biomarker and therapeutic target for CCA.

## Electronic supplementary material

Below is the link to the electronic supplementary material.

Supplementary Material 1

Supplementary Material 2

Supplementary Material 3

Supplementary Material 4

Supplementary Material 5

Supplementary Material 6

## Data Availability

The data that support the findings of this study are available from the corresponding author upon reasonable request. Raw RNA-seq data were uploaded to the GEO database (GSE275480).

## Abbreviations

CCA: Cholangiocarcinoma
UHRF1: Ubiquitin like with PHD and ring finger domains 1
DNMT1: DNA methyltransferase 1
PTMs: Posttranscriptional modifications
STUB1: STIP1 homology and U-Box containing protein 1
PLA2G2A: Phospholipase A2 group IIA
TSGs: Tumor suppressor genes
CRC: Colorectal cancer
CDS: Coding sequences
CCK-8: Cell Count Kit-8
RT-qPCR: Real time quantitative polymerase chain reaction
BSP: Bisulfite sequencing PCR
IHC: Immunohistochemistry
Co-IP: Co-immunoprecipitation
ChIP-seq: Chromatin immunoprecipitation sequence
DAC: Decitabine
HE: Hematoxylin-eosin
