# Correction: STUB1-mediated K63-linked ubiquitination of UHRF1 promotes the progression of cholangiocarcinoma by maintaining DNA hypermethylation of PLA2G2A

**DOI:** 10.1186/s13046-024-03198-2

**Published:** 2024-10-08

**Authors:** Junsheng Chen, Da Wang, Guanhua Wu, Fei Xiong, Wenzheng Liu, Qi Wang, Yiyang Kuai, Wenhua Huang, Yongqiang Qi, Bing Wang, Yongjun Chen

**Affiliations:** 1 Department of Biliary-Pancreatic Surgery, Tongji Hospital, Tongji Medical College, Huazhong University of Science and Technology, 1095 Jiefang Avenue, Wuhan, Hubei 430074 China; 2 Department of General Surgery, Beijing Friendship Hospital, Capital Medical University, Beijing, 100050 China; 3 Department of Emergency, Tongji Hospital, Tongji Medical College, Huazhong University of Science and Technology, 1095 Jiefang Avenue, Wuhan, Hubei 430074 China; 4 Key Laboratory of Laparoscopic Technology of Zhejiang Province, Department of General Surgery, Sir Run-Run Shaw Hospital, Zhejiang University School of Medicine, Hangzhou, 310016 China

**Correction:** ***J Exp Clin Cancer Res*** **43, 260 (2024)**

10.1186/s13046-024-03186-6.

Following publication of the original article [1], the authors found errors in the Supplementary Materials. Figure S1-S3 and their corresponding legends were mistakenly not processed and captured. Furthermore, the order of the efiles were incorrect and some efiles contain yellow highlights of the texts.

The Supplementary Materials are now corrected and updated.

These corrections do not affect the overall result or conclusion of the article. The original article [1] has been corrected.

## Electronic supplementary material

Below is the link to the electronic supplementary material.

Supplementary Material 1

Supplementary Material 2

Supplementary Material 3

Supplementary Material 4

Supplementary Material 5

Supplementary Material 6
