# Supplementary material for: STUB1-mediated K63-linked ubiquitination of UHRF1 promotes the progression of cholangiocarcinoma by maintaining DNA hypermethylation of PLA2G2A
Source: J Exp Clin Cancer Res. 2024 Sep 13;43:260. doi: 10.1186/s13046-024-03186-6 (PMC11395162; doi:10.1186/s13046-024-03186-6)
Supplement: Supplementary file 1 — Supplementary Material 1 [file 13046_2024_3186_MOESM1_ESM.docx]

Additional file 1. Primers for plasmid construction.

| Gene | Sequence |
| --- | --- |
| shSTUB1#1F | CCGGGACGCATTCATCTCTGAGAATCTCGAGATTCTCAGAGATGAATGCGTCTTTTTG |
| shSTUB1#1R | AATTCAAAAAGACGCATTCATCTCTGAGAATCTCGAGATTCTCAGAGATGAATGCGTC |
| shSTUB1#2F | CCGGGCAGTCTGTGAAGGCGCACTTCTCGAGAAGTGCGCCTTCACAGACTGCTTTTTG |
| shSTUB1#2R | AATTCAAAAAGCAGTCTGTGAAGGCGCACTTCTCGAGAAGTGCGCCTTCACAGACTGC |
| shDNMT1#1F | CCGGCCCGAGTATGCGCCCATATTTCTCGAGAAATATGGGCGCATACTCGGGTTTTTG |
| shDNMT1#1R | AATTCAAAAACCCGAGTATGCGCCCATATTTCTCGAGAAATATGGGCGCATACTCGGG |
| shDNMT1#2F | CCGGCGAGAAGAATATCGAACTCTTCTCGAGAAGAGTTCGATATTCTTCTCGTTTTTG |
| shDNMT1#2R | AATTCAAAAACGAGAAGAATATCGAACTCTTCTCGAGAAGAGTTCGATATTCTTCTCG |
| shPLA2G2A#1F | CCGGTGCTAGAAACAAGACGACCTACTCGAGTAGGTCGTCTTGTTTCTAGCATTTTTG |
| shPLA2G2A#1R | AATTCAAAAATGCTAGAAACAAGACGACCTACTCGAGTAGGTCGTCTTGTTTCTAGCA |
| shPLA2G2A#2F | CCGGGATCAAGTTGACGACAGGAAACTCGAGTTTCCTGTCGTCAACTTGATCTTTTTG |
| shPLA2G2A#2R | AATTCAAAAAGATCAAGTTGACGACAGGAAACTCGAGTTTCCTGTCGTCAACTTGATC |
| oeSTUB1-Flag-F | CGGATCGGGTTTAAACGGATCCGCCACCATGAAGGGCAAGGAGGAGAAG |
| oeSTUB1-Flag-R | GCCCTCTAGACTCGAGCGGCCGGTAGTCCTCCACCCAGCCATT |
| oeSTUB1-HA-F | CGGATCGGGTTTAAACGGATCCGCCACCATGAAGGGCAAGGAGGAGAAG |
| oeSTUB1-HA-R | GCCCTCTAGACTCGAGCGGCCGTCAGTAGTCCTCCACCCAGCC |
| oeRNF2-Flag-F | CGGATCGGGTTTAAACGGATCCGCCACCATGTCTCAGGCTGTGCAGACA |
| oeRNF2-Flag-R | GCCCTCTAGACTCGAGCGGCCGTTTGTGCTCCTTTGTAGGTGC |
| oeTRIM26-Flag-F | CGGATCGGGTTTAAACGGATCCGCCACCATGGCCACGTCAGCCCCACTA |
| oeTRIM26-Flag-R | GCCCTCTAGACTCGAGCGGCCGGGGTCTTAGCAGGAGGCGTGT |
| oeTRIP12-Flag-F | CGGATCGGGTTTAAACGGATCCGCCACCATGTTAATGGCCAAGGCTATCATG |
| oeTRIP12-Flag-R | GCCCTCTAGACTCGAGCGGCCGTCCAACAGGCAATCTTGGGCT |
| oeTRIP12-mid-Flag-F | AATTTAGAGGAGTATCTAAGACTGGATCAGCTCCTTTGTGGC |
| oeTRIP12-mid-Flag-R | TCTTAGATACTCCTCTAAATT |
| oeSTUB1-GST-F | GGATCTGGTTCCGCGTGGATCCATGAAGGGCAAGGAGGAGAAG |
| oeSTUB1-GST-R | AGTCACGATGCGGCCGCTCGAGTCAGTAGTCCTCCACCCAGCC |
| oeStub1-F | GTGTGGTGGAATTCTGCAGATAGCCACCATGAAGGGCAAGGAGGAAAAG |
| oeStub1-R | GATCAGCGGGTTTAAACCATGGTCAATAGTCCTCTACCCAGCC |
| oeUHRF1-Flag-F | CGGATCGGGTTTAAACGGATCCGCCACCATGTGGATCCAGGTTCGGACC |
| oeUHRF1-Flag-R | GCCCTCTAGACTCGAGCGGCCGCCGGCCATTGCCGTAGCCGGGG |
| oeUHRF1-HA-F | CGGATCGGGTTTAAACGGATCCGCCACCATGTGGATCCAGGTTCGGACC |
| oeUHRF1-HA-R | GCCCTCTAGACTCGAGCGGCCGTCACCGGCCATTGCCGTAGCC |
| oeUHRF1-GST-F | GGATCTGGTTCCGCGTGGATCCATGTGGATCCAGGTTCGGACC |
| oeUHRF1-GST-R | AGTCACGATGCGGCCGCTCGAGTCACCGGCCATTGCCGTAGCC |
| oeUhrf1-F | GTGTGGTGGAATTCTGCAGATAGCCACCATGTGGATCCAGGTTCGAACT |
| oeUhrf1-R | GATCAGCGGGTTTAAACCATGGTCACCGGCCGCTGCCATAGCC |
| oeDNMT1-F | CGGATCGGGTTTAAACGGATCCGCCACCATGCCGGCGCGTACCGCCCCA |
| oeDNMT1-R | GCCCTCTAGACTCGAGCGGCCGCTAGTCCTTAGCAGCTTCCTC |
| oePLA2G2A-F | CGGATCGGGTTTAAACGGATCCGCCACCATGAAGACCCTCCTACTGTTG |
| oePLA2G2A-R | GCCCTCTAGACTCGAGCGGCCGTCAGCAACGAGGGGTGCTCCC |
| STUB1-D1-Flag-F | GCGCAGGAGCTCAAGGAGCAGGG |
| STUB1-D1-Flag-R | CTGCTCCTTGAGCTCCTGCGCCATGGTGGCGGATCCGTTTAA |
| STUB1-D2-Flag-F | CGGCTGAACTTCGGGGACGAC |
| STUB1-D2-Flag-R | GTCGTCCCCGAAGTTCAGCCGGCTCGGGCTCTTCTCGGGGCTT |
| STUB1-D3-Flag-F | GACATCCCCGACTACCTGTGT |
| STUB1-D3-Flag-R | ACACAGGTAGTCGGGGATGTCCTGCTCCTTGGCCAGGCTGTA |
| STUB1-D4-Flag-F | CGGCCGCTCGAGTCTAGAGGG |
| STUB1-D4-Flag-R | CCCTCTAGACTCGAGCGGCCGTCGCTTCTTCCTCTTCTCATC |
| STUB1-O2-Flag-F | CGGATCGGGTTTAAACGGATCCGCCACCATGGCGCAGGAGCTCAAGGAGCAG |
| STUB1-O2-Flag-R | GCCCTCTAGACTCGAGCGGCCGCTGCTCCTTGGCCAGGCTGTA |
| UHRF1-D1-Flag-F | CTCCCCCACAGCACCAAGGAG |
| UHRF1-D1-Flag-R | CTCCTTGGTGCTGTGGGGGAGCATGGTGGCGGATCCGTTTAA |
| UHRF1-D2-Flag-F | GGGCTGTACAAGGTCAATGAG |
| UHRF1-D2-Flag-R | CTCATTGACCTTGTACAGCCCCACGAGGCTCTGGCGGACCAG |
| UHRF1-D3-Flag-F | GAAGGGAGCCCCATGGTTGAC |
| UHRF1-D3-Flag-R | GTCAACCATGGGGCTCCCTTCCAATTCCGTCTCATCCCACAT |
| UHRF1-D4-Flag-F | AACAGACTCTGCCGGGTCTGC |
| UHRF1-D4-Flag-R | GCAGACCCGGCAGAGTCTGTTACCCGGCCGCTCAATCTTGAA |
| UHRF1-D5-Flag-F | GCCAGCGAGGTGGTACTGGCG |
| UHRF1-D5-Flag-R | CGCCAGTACCACCTCGCTGGCCACGTCGTCCTTGCAGTGCTT |
| UHRF1-D6-Flag-F | CACTACGGACCCATCCCGGGG |
| UHRF1-D6-Flag-R | CCCCGGGATGGGTCCGTAGTGATCATTCCGGCACTCAGGGCA |
| UHRF1-D7-Flag-F | GATGAGCCTGGCCCTTGGACG |
| UHRF1-D7-Flag-R | CGTCCAAGGGCCAGGCTCATCGTTGGACGGGACGATGGTACA |
| UHRF1-D8-Flag-F | TGTATCTGCTGTCAGGAGCTG |
| UHRF1-D8-Flag-R | CAGCTCCTGACAGCAGATACAATCGTCCCTCCGCAGAAGGTA |
| UHRF1-D9-Flag-F | CGGCCGCTCGAGTCTAGAGGG |
| UHRF1-D9-Flag-R | CCCTCTAGACTCGAGCGGCCGCTGGAACGTCTCCTCCACTTT |
| UHRF1-O7-Flag-F | CGGATCGGGTTTAAACGGATCCGCCACCATGCACTACGGACCCATCCCGGGG |
| UHRF1-O7-Flag-R | GCCCTCTAGACTCGAGCGGCCGATCGTCCCTCCGCAGAAGGTA |
| UHRF1-O9-Flag-F | CGGATCGGGTTTAAACGGATCCGCCACCATGTGTATCTGCTGTCAGGAGCTG |
| UHRF1-O9-Flag-R | GCCCTCTAGACTCGAGCGGCCGCCGGCCATTGCCGTAGCCGGG |
