# Supplementary material for: STUB1-mediated K63-linked ubiquitination of UHRF1 promotes the progression of cholangiocarcinoma by maintaining DNA hypermethylation of PLA2G2A
Source: J Exp Clin Cancer Res. 2024 Sep 13;43:260. doi: 10.1186/s13046-024-03186-6 (PMC11395162; doi:10.1186/s13046-024-03186-6)
Supplement: Supplementary file 2 — Supplementary Material 2 [file 13046_2024_3186_MOESM2_ESM.docx]

Additional file 2. Primary antibodies used in this study.

| Target | Host | ID | Supplier | Application |
| --- | --- | --- | --- | --- |
| HRP-Flag | Mouse | HRP-66008 | Proteintech | WB |
| HRP-HA | Rabbit | HRP-81290 | Proteintech | WB |
| Flag | Mouse | 66008-3-Ig | Proteintech | IP |
| HA | Mouse | AE008 | ABclonal | IP |
| STUB1 | Rabbit | ab134064 | Abcam | WB,IP,IHC |
| STUB1 | Mouse | 68407-1-Ig | Proteintech | WB |
| UHRF1 | Rabbit | 21402-1-AP | Proteintech | WB,IP,IF,IHC |
| UHRF1 | Mouse | ab57083 | Abcam | WB |
| GST | Mouse | 66001-2-Ig | Proteintech | WB,IP |
| GST | Rabbit | 10000-0-AP | Proteintech | WB,IP |
| MYC | Rabbit | 16286-1-AP | Proteintech | WB |
| GAPDH | Rabbit | BM3874 | BOSTER | WB |
| DNMT1 | Rabbit | 5032S | Cell Signaling Technology | WB |
| Histone H3 | Rabbit | ab176842 | Abcam | WB |
| Tublin | Mouse | 66031-1-Ig | Proteintech | IF |
| Rabbit IgG | Rabbit | 30000-0-AP | Proteintech | IP |
| Mouse IgG | Mouse | B900620 | Proteintech | IP |
| PLA2G2A | Rabbit | ab23705 | Abcam | WB,IHC |
