# Supplementary material for: STUB1-mediated K63-linked ubiquitination of UHRF1 promotes the progression of cholangiocarcinoma by maintaining DNA hypermethylation of PLA2G2A
Source: J Exp Clin Cancer Res. 2024 Sep 13;43:260. doi: 10.1186/s13046-024-03186-6 (PMC11395162; doi:10.1186/s13046-024-03186-6)
Supplement: Supplementary file 4 — Supplementary Material 4 [file 13046_2024_3186_MOESM4_ESM.docx]

Additional file 4. Primers for RT-qPCR.

| Gene | Sequence |
| --- | --- |
| GAPDH-F | GGAGCGAGATCCCTCCAAAAT |
| GAPDH-R | GGCTGTTGTCATACTTCTCATGG |
| STUB1-F | AGCAGGGCAATCGTCTGTTC |
| STUB1-R | CAAGGCCCGGTTGGTGTAATA |
| UHRF1-F | GCCATACCCTCTTCGACTACG |
| UHRF1-R | GCCCCAATTCCGTCTCATCC |
| DNMT1-F | CCTAGCCCCAGGATTACAAGG |
| DNMT1-R | ACTCATCCGATTTGGCTCTTTC |
| PLA2G2A-F | ATGAAGACCCTCCTACTGTTGG |
| PLA2G2A-R | GCTTCCTTTCCTGTCGTCAACT |
| RFX6-F | AAGCAGCGGATCAATACCTGT |
| RFX6-R | ACCGTGGTAAGCAAACTCCTT |
| SLC1A6-F | ACAGTTCAAGACGCAGTACAG |
| SLC1A6-R | CCAAGGCCCGAGTGACATTTT |
| MYCN-F | ACCCGGACGAAGATGACTTCT |
| MYCN-R | CAGCTCGTTCTCAAGCAGCAT |
| MAPK4-F | TGAGAAGGGTGACTGCATCG |
| MAPK4-R | ACCAAACCATTGACACCGAAG |
| UGT1A9-F | CCCCCTTCCTCTATGTGTGTG |
| UGT1A9-R | TCATACTCCGTAACAGGTGTTTG |
| RELN-F | CAACCCCACCTACTACGTTCC |
| RELN-R | TCACCAGCAAGCCGTCAAAAA |
| GABRA2-F | GCTGGCTAACATCCAAGAAGAT |
| GABRA2-R | GCCGATTATCGTAACCATCCAGA |
| DEFB1-F | ATGAGAACTTCCTACCTTCTGCT |
| DEFB1-R | TCTGTAACAGGTGCCTTGAATTT |
| LRFN5-F | ATCTGTCCAAAGCGTTGTGTC |
| LRFN5-R | TGGTGGAACAAATAAAAGCCCT |
