# Supplementary material for: STUB1-mediated K63-linked ubiquitination of UHRF1 promotes the progression of cholangiocarcinoma by maintaining DNA hypermethylation of PLA2G2A
Source: J Exp Clin Cancer Res. 2024 Sep 13;43:260. doi: 10.1186/s13046-024-03186-6 (PMC11395162; doi:10.1186/s13046-024-03186-6)
Supplement: Supplementary file 5 — Supplementary Material 5 [file 13046_2024_3186_MOESM5_ESM.docx]

Additional file 5. Primers for Bisulfite sequencing PCR (BSP).

| Gene | Sequence |
| --- | --- |
| PLA2G2A-F | GGGTTATTTAGATATATAGAGAGGGAGG |
| PLA2G2A-R | ACAAACTAACACAACCTCCAAAAAT |
