# Supplementary material for: STUB1-mediated K63-linked ubiquitination of UHRF1 promotes the progression of cholangiocarcinoma by maintaining DNA hypermethylation of PLA2G2A
Source: J Exp Clin Cancer Res. 2024 Sep 13;43:260. doi: 10.1186/s13046-024-03186-6 (PMC11395162; doi:10.1186/s13046-024-03186-6)
Supplement: Supplementary file 6 — Supplementary Material 6 [file 13046_2024_3186_MOESM6_ESM.zip › Additional File 6/Supplementary Figures legend.docx]

**Figure S1. Further validation of ubiquitin proteins that may interact with UHRF1 in mass spectrometry results.**

**A-C.** Co-immunoprecipitation assays using Flag/HA antibody in HEK293T cells and immunoblotting to analyze the interaction of exogenous RNF2 (A), TRIP12 (B), TRIM26 (C) with exogenous UHRF1. And the only combination observed is between TRIM26 and UHRF1. **D.** TRIM26 (Flag-tagged), ubiquitin (Myc-tagged), and UHRF1 (HA-tagged) plasmids were exogenously overexpressed in HEK293T cells. Ubiquitination assays were conducted 48 h post-transfection, and the ubiquitination of UHRF1 was analyzed using a denaturing-immunoprecipitation assay.

**Figure S2. STUB1-UHRF1/DNMT1 axis promotes epigenetic silencing of PLA2G2A in CCA cells.**

1. The relative mRNA levels of top 10 genes among the downregulated group were validated by RT-qPCR assays. **B, D.** RNA was extracted from HuCCT1 cells with stable knockdown and overexpression of STUB1 (B) and DNMT1 (D) for RT-qPCR analysis to assess mRNA levels of PLA2G2A. **C**，**E.** Proteins were extracted from HuCCT1 cells with stable knockdown and overexpression of STUB1 (C) and DNMT1 (E) for Western blotting assays to assess the protein levels of PLA2G2A. **F, G.** The mRNA and protein expression of PLA2G2A in HuCCT1 cells treated with DAC were analyzed using RT-qPCR (F) and Western blotting (G) assays.

**Figure S3. Expression patterns of UHRF1 and PLA2G2A in xenograft tumor model and mouse primary CCA model.**

**A.** The expression of UHRF1 (upper channel) and PLA2G2A (down channel) in the xenograft tumor model was analyzed using IHC assays, with representative graphs displayed on the left and statistical analysis presented on the right. The staining intensities of the cytoplasm and nucleus were compared to determine the cytoplasmic: nuclear ratio as a relative measure of UHRF1 nuclear localization. **B.** The expression of UHRF1 (upper channel) and PLA2G2A (down channel) in the mouse primary CCA model was analyzed using IHC assays, with representative graphs displayed on the left and statistical analysis presented on the right.
