# Supplementary figures and images for: STUB1-mediated K63-linked ubiquitination of UHRF1 promotes the progression of cholangiocarcinoma by maintaining DNA hypermethylation of PLA2G2A
Source: J Exp Clin Cancer Res. 2024 Sep 13;43:260. doi: 10.1186/s13046-024-03186-6 (PMC11395162; doi:10.1186/s13046-024-03186-6)

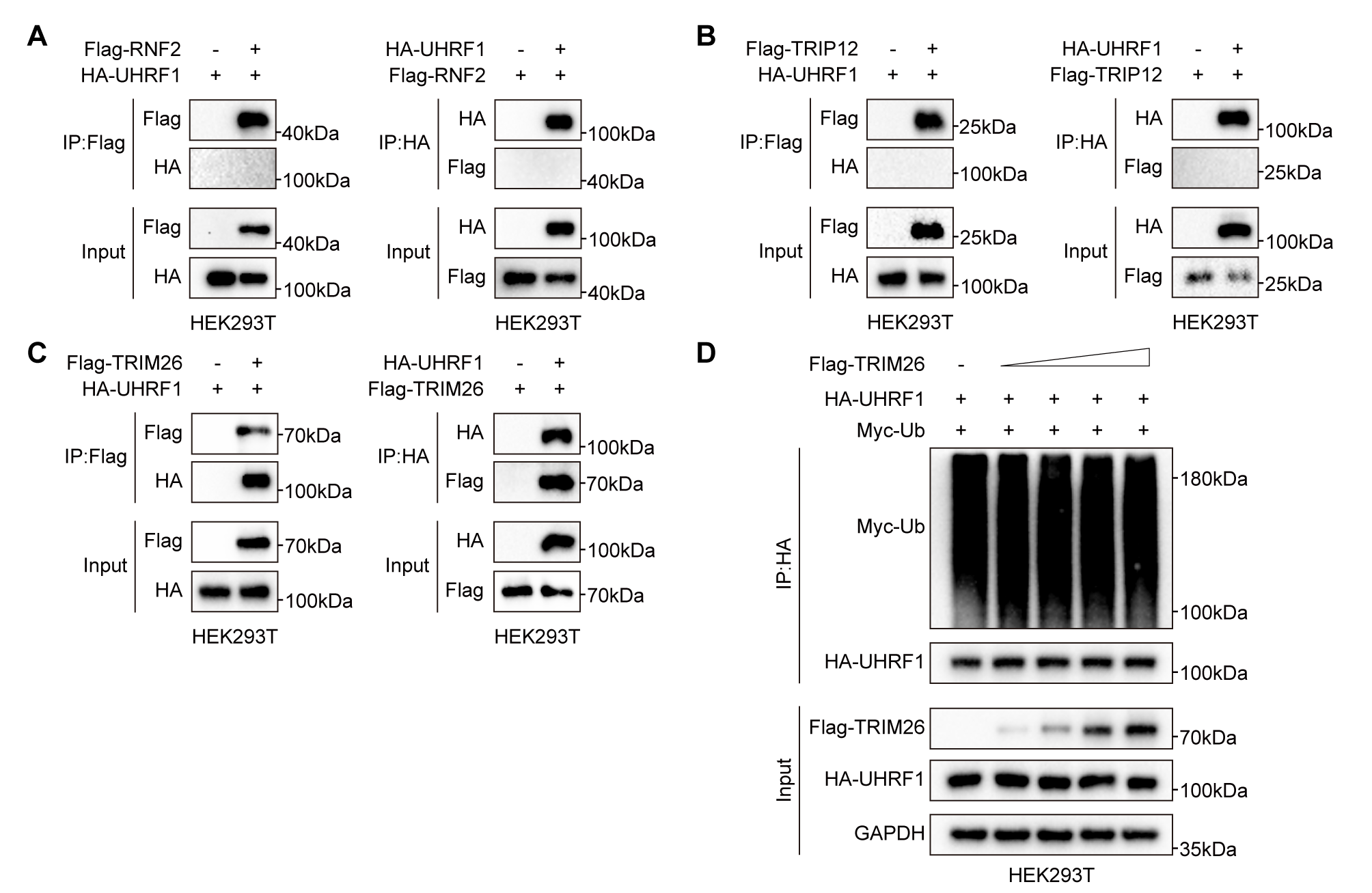

Supplement: Supplementary file 6 — Supplementary Material 6 [file 13046_2024_3186_MOESM6_ESM.zip › Additional File 6/Figure S1.tif]

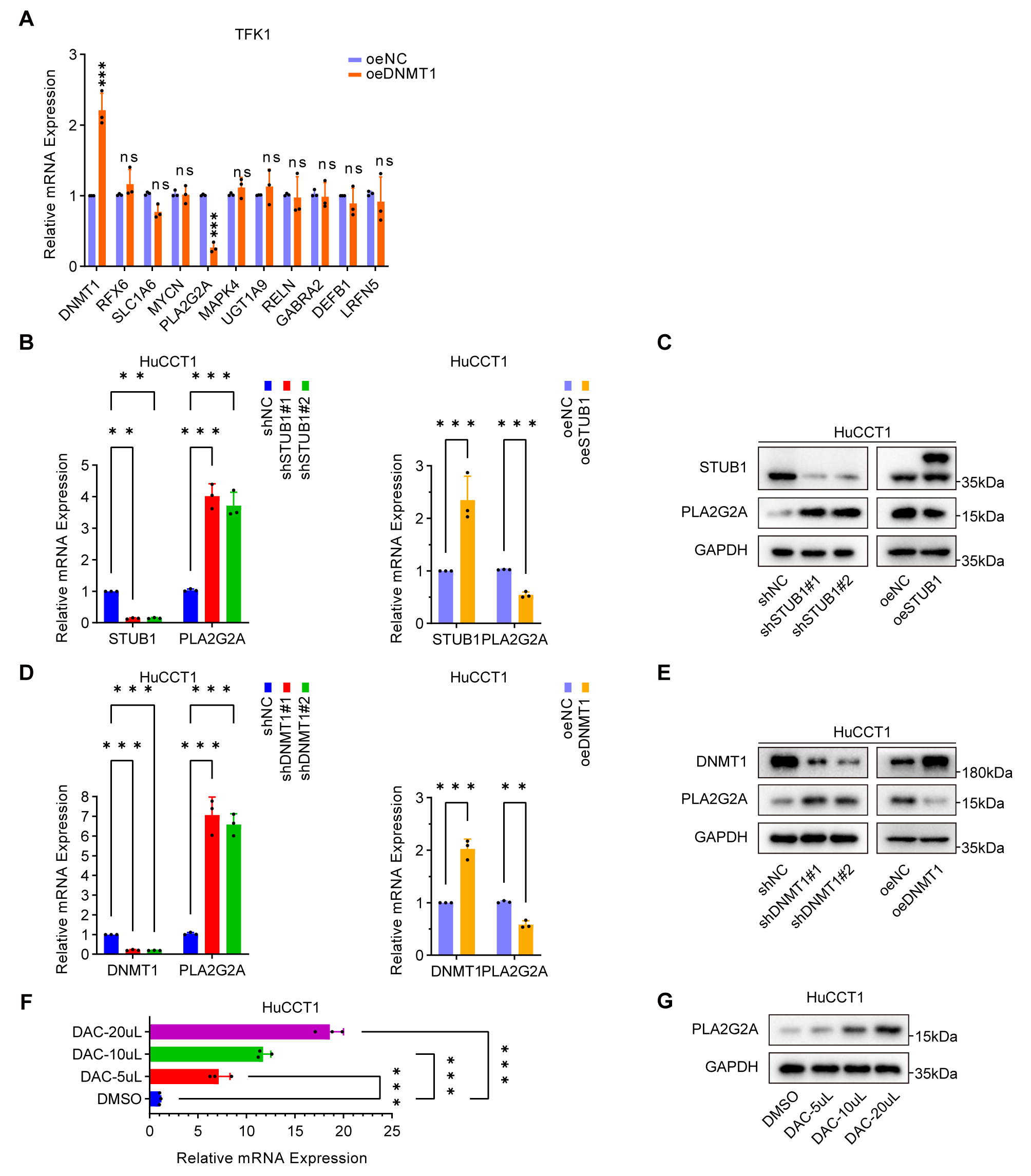

Supplement: Supplementary file 6 — Supplementary Material 6 [file 13046_2024_3186_MOESM6_ESM.zip › Additional File 6/Figure S2.tif]

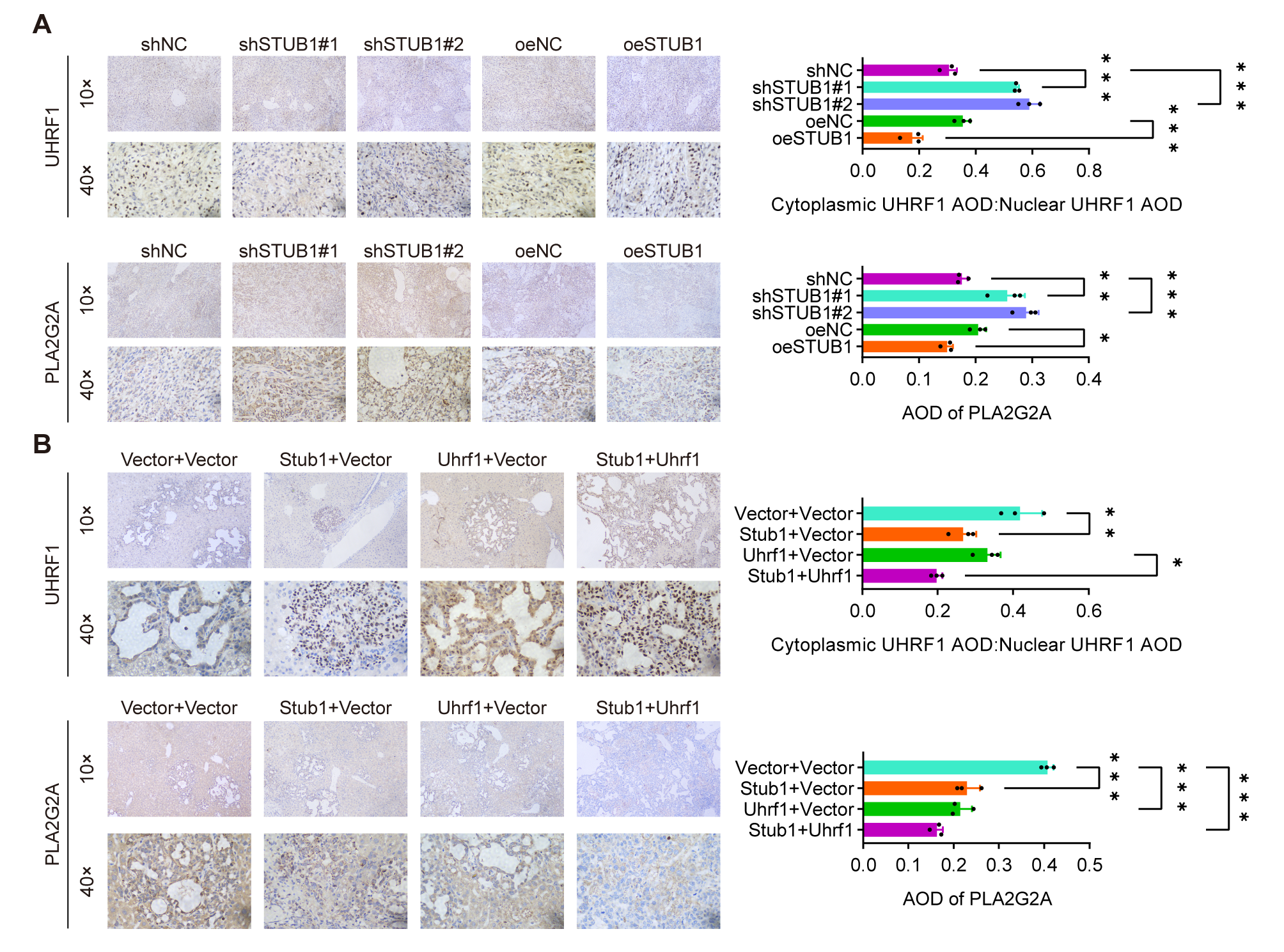

Supplement: Supplementary file 6 — Supplementary Material 6 [file 13046_2024_3186_MOESM6_ESM.zip › Additional File 6/Figure S3.tif]
